# Bio-Guided Assay of *Ephedra foeminea* Forssk Extracts and Anticancer Activities: In Vivo, In Vitro, and In Silico Evaluations

**DOI:** 10.3390/molecules29010199

**Published:** 2023-12-29

**Authors:** Jilan A. Nazeam, Sylvia A. Boshra, Esraa Z. Mohammed, Heba A. El Gizawy

**Affiliations:** 1 Pharmacognosy Department, Faculty of Pharmacy, October 6 University, 6th of October City 12585, Egypt; jilannazeam@o6u.edu.eg; 2 Biochemistry Department, Faculty of Pharmacy, October 6 University, 6th of October City 12585, Egypt; sylviaazmy@o6u.edu.eg; 3 Pharmaceutical Chemistry Department, Faculty of Pharmacy, October 6 University, 6th of October City 12585, Egypt; dr.esraazakaria@o6u.edu.eg

**Keywords:** *Ephedra foeminea*, alkaloids, proanthocyanidins, 6-methoxykynurenic acid, tumor marker, in silico, ADME

## Abstract

*Ephedra* is one of the oldest known medicinal plants and the largest genera of the Ephedraceae family. In vivo antitumor evaluation of *Ephedra foeminea* revealed that ethyl acetate (EtOAc) was the most bioactive fraction. Bio-guided fractionation of EtOAc fraction afforded nine compounds isolated for the first time from the plant species. Macrocyclic spermine alkaloids (1,9), proanthocyanidins (2,4,5), quinoline alkaloids (7,8), phenolic (3), and nucleoside (6) were identified and elucidated by spectroscopic analyses including 1D and 2D NMR, ESI-MS-MS spectrometry. The tested compounds exhibited moderate anticancer activity, except for the kynurenic acid derivative (6-mKYNA) which showed significant cytotoxicity and remarkable inhibition of CA-19.9 and CA-125 tumor biomarkers. In-silico study was conducted to determine the anti-proliferative mechanism of 6-mKYNA by using the CK2 enzyme active site. Moreover, the ADME computational study suggested that 6-mKYNA is an effective candidate with a promising pharmacokinetic profile and therapeutic potential against various types of cancer.

## 1. Introduction

The genus *Ephedra* (Ephedraceae) is widely distributed worldwide, and is considered one of the oldest medicinal plants [1]. Traditionally, *Ephedra* was used to treat lung diseases (allergies, bronchial asthma, colds, and coughs), headaches, nasal congestion, edema, fever, and other common diseases [2]. Antidiabetic, anti-obesity, antiviral, and antitumor activities have also been reported [3]. Moreover, *Ephedra* herb is the main component of different pharmaceutical dosage forms, including ordinary tablets, chewable tablets, capsules, syrups, and drops [4].

Previous phytochemical analyses have revealed that more than 145 compounds have been isolated and identified from the *Ephedra* genus, including alkaloids [5,6,7], flavonoids [8,9,10], proanthocyanidins, lignans, polyphenols [11], and polysaccharides [12,13]. Nevertheless, the reported data indicated that most of the isolated constituents from the different species of *Ephedra* have not been pharmacologically assessed [14]. Indeed, the wide geographic distribution of *Ephedra* species could influence the morphological characteristics and variations of secondary metabolites, which have not been fully investigated [14].

The use of *Ephedra* has been restricted because of its potential side effects [15]. The aerial parts of different *Ephedra* species contain phenylpropylamino-active sympathomimetic alkaloids, which could lead to an increase in heart contractility as well as overstimulation of the central nervous system. However, *Ephedra foeminea* Forssk species uniquely lack both ephedrine and pseudoephedrine [16,17].

In recent years, *E. foeminea* has gained interest as a traditional alternative cancer treatment [18]. According to Mendelovich et al. (2017), the ethanolic extract of the leaves and fruit juice possesses a cytotoxic effect against colon (HTC116) and breast cancer cells (MDA-MB-213), with a caspase-3-dependent apoptosis induction effect [17]. However, there are few reports on the chemical composition of plants and their related pharmacological activities [13]. Therefore, it was deemed of interest to explore its phytochemical constituents with respect to its anticancer activity.

In this study, an in vivo study was conducted using a bio-guided fractionation approach. Different compounds were isolated from most bioactive fractions and elucidated. The cytotoxic activity of the compounds was assessed against breast cancer cell lines (MCF-7). The molecular activity of the most active compound was evaluated using tumor biomarkers CA-19.9 and CA-125. In addition, an in silico study and ADME analysis predicted the mechanism of action and pharmacokinetic profile of *Ephedra* bioactive compounds.

## 2. Results and Discussion

### 2.1. In Vivo Antitumor Activity against Ehrlich Ascites Carcinoma

Recent reports have indicated that *Ephedra* herb could be used to treat various cancer types. However, there is insufficient characterization of its pharmacological activity and relevant mechanism, especially in *E. foeminea* [19]. Fractionation of the dichloromethane extract of aerial plant parts afforded four fractions: hexane, dimethyl chloride (CH_2_Cl_2_), ethyl acetate (EtOAc), and methanol (MeOH).

Ehrlich ascites carcinoma (EAC) is an experimental breast tumor derived from a spontaneous mouse adenocarcinoma. Ehrlich ascites tumor implantation induces a local inflammatory reaction with increased vascular permeability. The resulting intense edema, cellular migration, and progressive ascitic fluid formation play important roles in tumor growth [20]. After oral administration of the *Ephedra* fractions, a significant decrease (*p* < 0.05) in tumor weight and volume was observed compared to the EAC-bearing mice. The results indicate a decrease in abnormal cell divisions and tumor proliferation; hence, they could revert EAC-induced tumor action [21].

The ethyl acetate (EtOAc) fraction showed the most significant activity (Table 1). Tumor-associated CA-19.9 and CA-125 antigens have been accepted as the most meaningful tumor markers [22]. In the present study, EAC-bearing mice showed a significant increase in the serum levels of CA-19.9 and CA–125, owing to the metastatic properties of the EAC cells that can induce a variety of abdominal tumors, including breast, pancreatic, ovarian, gastric, and colorectal tumors [23].

**Figure 1:**
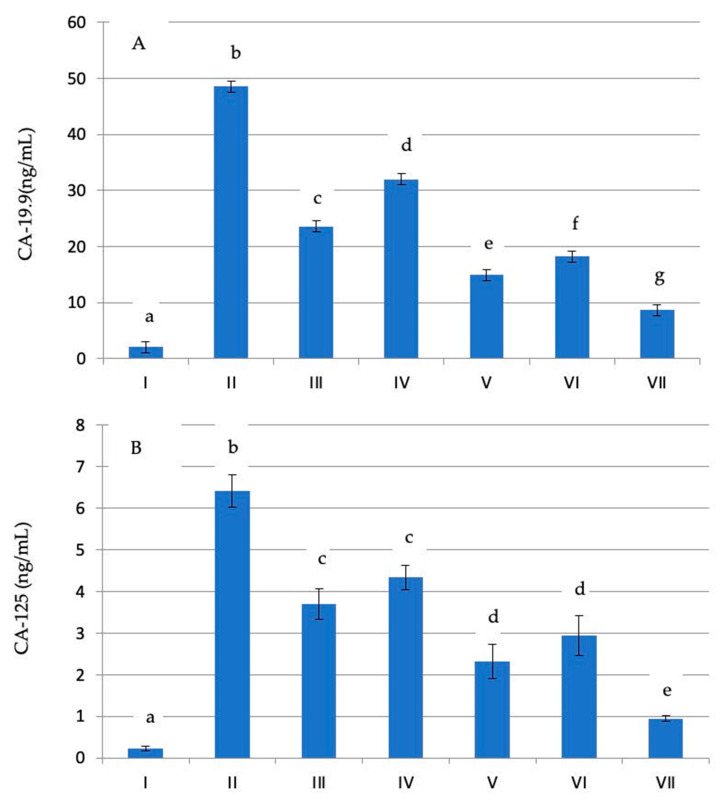
Effect of *E. foeminea* Forssk fractions (hexane, dichloromethane, ethyl acetate, and methanol fractions) and 5FU on plasma CA-19.9 (**A**) and CA-125 (**B**) in the normal group (I) and Ehrlich ascites carcinoma-bearing mice groups (II–VII). Letters (a–g) indicate significant differences at *p* ≤ 0.05. Data followed by the same letter are not significantly different at *p* ≤ 0.05.

As shown in Figure 1a,b, the plasma CA-19.9 and CA-125 levels were elevated in association with an increase in the tumor volume and weight in the group bearing EAC when compared with the normal control group (*p* < 0.05). Treatment of mice with *Ephedra* fractions resulted in a significant decrease in plasma CA-19.9 and CA-125 levels. The effect of the ethyl acetate (EtOAc) fraction was more pronounced than that of the other fractions, with a lower doublet than that of EAC.

### 2.2. Isolation of Compounds from the (EtOAc) Fraction

Plants are considered a good source of chemical diversity for drug discovery [24,25]. Different classes of chemical constituents were isolated from EtOAc, as it is the most bioactive fraction containing macrocyclic spermine alkaloids, quinoline alkaloids, proanthocyanidins, phenols, and nucleosides. In the last two decades, ESI-MS_2_ has been one of the most sensitive and quick methods for the characterization of plant metabolites [26,27].

#### 2.2.1. Macrocyclic Spermine Alkaloids Isolated Compounds

Alkaloids were the first active ingredients isolated from the *Ephedra* herb and have been used as a standard for evaluating the quality of *Ephedra* species [28,29]. Compounds **1** and **9** showed a characteristic signal at *m*/*z* 459.2 for [C_30_H_39_N_2_O_2_^.^], which is a characteristic of macrocyclic spermine alkaloids (Supplementary Material; Figure S1).

Compound **1**, a negative ESI-MS/MS fragmentation pattern, was similar to that of ephedradine A (C_28_H_36_N_4_O_4_, 492.2), with an existing molecular ion peak MS_2_ [M–CO-2H] ion at *m*/*z* 461.3. The fragmentation pattern of compound **9** was matched with literature data for ephedradine B (C_29_H_38_N_4_O_5_, 522.2), exhibiting a molecular ion peak at *m*/*z* 503.3; [C_29_H_35_N_4_O_4_^2.^] represents [M-H_2_O-H]. The dihydrobenzofuran nucleus, which bridges a seventeen-membered lactam ring containing the spermine unit of the ephedradine moiety, was recognized from the ^1^H and ^13^C nuclear magnetic resonance (NMR) and HSQC spectral data for compounds **1** and **9** (Table 2) (Supplementary Material; Figures S10–S12) and (Supplementary Material; Figures S34–S36) respectively. The presence of the spermine moiety for both compounds was attributed to the remarkable multiple shielded protons in a range from 1.2 to 3.4 that were identified to represent 12 aliphatic protons (11 × CH_2_ and 1 CH). Some of these protons are attached to the N atoms, as indicated by their chemical shifts. 

The HSQC indicates that the spermine part was connected to the dihydrobenzofuran at H3/C3, which was correlated at 4.1/59.5 ppm for compound **1** (ephedradine A) and 4.7/49.9 ppm through the amide carbonyl represented at APT ^13^C NMR at 177.5. The downfield shift of the C-17/H-17 methin at 64/4.0 ppm for compound **1** indicates that it was flanked between an aromatic ring and N atom [30]. Consequently, C-17 was the second attachment site between the spermine moiety and the dihydrobenzofuran. The oxygenated C2 and C3 methine in the HSQC were all diagnostic for dihydrobenzofuran substitution. The downfield shift of H-2/C2 and H-3/C3 around 4.9/113.1 and 4.10/59.5 for ephedradine A (C1) and B (C10) referred to phenyl and carbonyl substitutions, respectively, while the large coupling constant (J = 11–12 ppm) was diagnostic for the trans-orientation of the two substituents [31].

The chemical shift of the six phenyl carbons includes one quaternary oxygenated carbon at 150.1 ppm for ephedradine A and two quaternary oxygenated carbons at 149.1 and 148.2 ppm for Ephedradine B, while one of these positions was occupied by a methoxy group at chemical shifts 3.60 and 65 in ^1^H-NMR and ^13^C-NMR, respectively. The remaining two oxygen atoms in the molecule are involved in two carbonyl groups that both are involved in an amide linkage with a remarkable signal at 169.10 and 169.11 ppm for ephedradine A and 177.5 and 178.0 ppm for ephedradine B [30]. Spermine alkaloids were isolated earlier from the roots of *Ephedra aphylla* and *Ephedra sinica* [32,33]. Previous studies have indicated that ephedradines exert an antihypertensive effect [31,34].

#### 2.2.2. Quinoline Alkaloids Isolated Compounds

Compound **7** was obtained as a pinkish powder and was identified as 6-methoxy knurenic acid (C_11_H_9_NO_4_, 219.0) in the negative ion mode of ESI-MS/MS, indicating a molecular ion peak at *m*/*z* 199.07 [M-H_2_O-2H], and the base peak at *m*/*z* 124.9 refers to the fragment ion [C_9_H_3_N^4.^] (Supplementary Material; Figure S7). The same later fragment represents the base peak for compound **9** (white amorphous powder) with a different diagnostic ion peak *m*/*z* 201.7, which refers to [M-2H-2H] for (C_10_H_7_NO_4_; 205.0) as 6-hydroxykynurenic acid. ESI-MS/MS revealed that compounds **8** and **9** are quinoline alkaloids (Supplementary Material; Figures S8 and S9).

For compounds **7** and **8**, structural characterization was achieved using 1D and 2D NMR analyses (Table 2). The spectrum displayed dominant resonances of five hydrogens at 7.03 ppm (s), 6.90 (s), 7.41 (d), 8.01 (d), and 3.81 (brs) with integral values in a quantitative ratio of 1:1:1:1:3. The ^13^C-NMR and HSQC spectra showed resonances of 12 carbons. Moreover, ^13^C-NMR showed characteristic signals at 174.6 and 50.5°, which were attributed to C12 and methoxy carbon, respectively. The physical and spectral data for compound **7** are in accordance with those reported in the literature for 4-hydroxy-6-methoxyquinoline-2-carboxylic acid (6-methoxy kynurenic acid) [35].

From the ^1^H and ^13^C NMR spectra, compound **8** was identified as 6-hydroxykynurenic acid and showed similarity to 6-methoxy-kynurenic acid, except that it lacked the methoxy carbon linked to the aromatic ring. Extensive ESI-MS/MS and HSQC analyses defined the chemical structure of compound **8**, which was differentiated from compound **7** by the presence of a hydroxy group linked to C6 of the quinoline moiety (Supplementary Material; Figures S28–S33). 6-hydroxykynurenic acid was reported as the major alkaloid in *E. foeminea* and *E. foliata,* whereas 6-methoxykynurenic acid was previously isolated from *E. pachyclada* [35].

#### 2.2.3. Proanthocyanidins Isolated Compounds

The ESI-MS/MS positive ion modes of compounds **2**, **4**, and **5** were characterized by a common ion, *m*/*z* 265.2, 338.3, and 563.6, which are attributed to the [C_18_H_17_O_2_^.^], [C_18_H_18_O_3_] (100%), [C_22_H_10_O_4_^8.^], and [C_30_H_11_O_12_^4.^] fragments, respectively. ESI-MS/MS, 1D, and 2D HSQC experiments enabled the complete identification of the structures. All of the data suggests that it belongs to the group of catechins/proanthocyanidins [36], where [M + Na]; 951.8 (calculated for C_45_H_36_O_2_, 928.0) of compound **2** (ephedrannin Tr5) moiety (Supplementary Material; Figure S2), [M + Na + H]; 564.5, (calculated for C_30_H_20_O_10_, 540.0) of compound **4** (ephedrannin B) (Supplementary Material; Figure S4), while [M + CO-H]; 619.5, (calculated for C_30_H_24_O_13_, 592.0) of compound **5** (ephedrannin D1) (Supplementary Material; Figure S5).

Compound **2** was obtained as an amorphous, white powder. ESI-MS/MS, together with the ^1^H NMR spectrum, ^13^C APT-NMR, and HSQC, indicated that compound **2** is a trimeric proanthocyanidin. The HSQC signals were assigned to two similar environments, H4′/C4′ of ring F (33.1/3.8) and C4/H4 of ring C, and the ^13^C-APT spectrum revealed signals for three 3′,4′,5′-trisubstituted flavan-3-ol moieties, indicating the presence of three gallocatechin groups. Moreover, three broad singlets at δ 130.0/7.61, 130.6/7.62, and 131.2/7.43, ascribed to C2′/H2′ in rings B, E, and H, respectively, revealed the presence of a trimeric moiety (Table 3). The spectra indicated two sets of characteristic correlations for C2′/H2′, C3′/H3′, and C4′/H4′ in-ring F (epigallocatechin residue) and ring I (gallocatechin residue): (71.2/4.37, d, *J* = 7.3 Hz), (114.2/4.9, m), and (56.1/3.8, d, *J* = 2.5 Hz), (71.2/4.37, d, *J* = 9.4 Hz), (68.1/4.15, m), and (37/2.2,1.8, d, *J* = 8.5, 16.3 Hz), respectively. In addition, the coupling constant of F-ring H-4 (*J* = 2.5 Hz) was consistent with the 3,4-trans configuration [37]. The ^1^H NMR singlet signal with correlation C6″/H6″ 130.1/5.2 in ring G also showed great similarity with the NMR data previously reported for ephedrannin Tr5 [38,39], (Supplementary Material; Figures S13–S15).

Compound **4** was obtained as a yellow, amorphous powder. The ^1^H- and ^13^C-NMR data closely resembles those of **2**, and **5** with a characteristic singlet signal of H6′/C6′ in ring D for all compounds represented as 7.75/79.5 (ephedrannin B), 5.2/130.5 (ephedrannin D1), and 5.0/115.2 (ephedrannin tr5). The HSQC spectra revealed the presence of characteristic signals in the dihydropyran ring of C4/H4 at 67.0/4.2 (t), the meta-coupled doublets at methylene C3/H3a, H3b 35/2.3, 2.4 (d, *J* = 4,12 Hz), in ring C, aromatic protons of ring A; H6/C6;4.8/79, H8/C8; 6.7/78 (brs), and the doubly linked dimeric structure was also demonstrated by an acetal carbon at C2 of ring C at δ113.7 in the ^13^C NMR spectrum and two AA′BB′ systems in the aromatic region (δ H3′/C3′, H5′/C5′; 6.42/114.9, 6.42/114.9, H2′/C2′, H6′/C6′; 6.60/138.2, 6.60/127.5) d, *J* = 8.5 Hz, due to rings B and (δ H2′/C2′, H6′/C6′ (6.63/130.5), (7.67/138.2), H3′/C3′ (6.65/115.8), H5’/C5’ (6.41/114.7), 2H (d, *J* = 8.0 Hz) of ring E confirmed the A-type dimeric proanthocyanidin structure. The NMR data mentioned above, together with the mass spectrometric information, implied that the structure of this proanthocyanidin, designated as ephedrannin B, was proposed to be 5,7,4′-trihydroxyflavan-[4(α) → 8,2(α) → O → 7]-kaempferol, which was previously reported in the roots of *Ephedra sinica* [40] (Supplementary Material; Figures S19–S21). Ephedrannin B was previously isolated from the roots of *E. sinica*, and exerts anti-inflammatory action [41], anti-hydrotic [42] and antimicrobial activities, and depigmentation effects on the skin [42].

Compound **5**, an amorphous white powder, showed a C-2 ketal carbon ^13^C NMR spectra at δC 112.0, suggesting that it was related to the A-type proanthocyanidin. The HSQC characteristic signal of C3/H3 and C4/H4 of ring C, 77.7/7.1 (1H, d, *J* = 3.5 Hz), and 26.5/2.6 (1H, d, *J* = 3.5 Hz) in ring C. Four characteristic singlet proton signals of ring B (H2′, H6′) and H6 and H8 of ring A indicate the presence of a gallocatechin group. The NMR data (Table 3) show great similarity with previously reported NMR data for (+)-epigallocatechin- (2α → O → 7,4α → 8) -(-)-catechin, a dimer, and was named ephedrannin D1 [35]. Compounds **5** and **2** (ephedrannin D1, and Tr5) have similar broad singlet signals with the same environment in ring E C2′/H2′ 130.6/7.4 (D1), and 130.6/7.62 (Tr5). Moreover, the C3 in ring C, saturated carbon C2′, and methene carbon C4′ of ring F could characterize ephedrannin D1 and Tr5 from ephedrannin B. There are D1 distinguishing signals that are absent in Tr5: free carbons in ring E; C2′/H2′, C5′/H5′, and C6′/H6′ 130.6/7.4 (brs), 112.3/6.7 (d, *J* = 8.1 Hz), and 130.1/7.3 (d, *J* = 8.1 Hz), respectively (Table 3) (Supplementary Material; Figures S22–S24).

#### 2.2.4. Phenol Isolated Compound

Compound **3** was obtained as a buff amorphous powder, and the ESI-MS/MS in the negative mode showed a fragmentation pattern analogous to phenolic nebrodenside (C_17_H_24_O_7_, 340.0) with (*m*/*z*, rel. int.): 399.2 [M-H], [C_17_H_23_O_7_]; 339.2, [C_11_H_14_O_2_]; 178.7, [C_11_H_12_O_2_^2.^]; 144.9. The ^1^H spectra revealed the presence of two allylic protons, one vinylic proton, and two vinylic methyl groups, indicating the presence of an isopent-2-enyl group (Table 3), (Supplementary Material; Figure S3).

In combination with the ^13^C NMR and HSQC spectra of compound **3**, 17 carbon resonances were observed for two methyl groups (^δ^C 15.0, 25.4), six carbons of glucopyranosyl ring, and six aromatic carbon signals were evident, two of which were oxygenated and indicated at 153.1 and 157.0, suggesting the presence of one benzene ring. Additionally, in the HSQC spectrum, the correlation and coupling patterns of the aromatic protons 95.9/7.26 (s), 96.2/7.23 (d, *J* = 8.4 Hz), and 78.8/7.2 (d, *J* = 8.7, 2.4 Hz) for at C2/H2, C5/H5, and C6/H6, respectively, suggest the presence of a 1,3,4-trisubstituted benzene ring. The correlation between the anomeric proton and carbon C1″/H1″ at 85.8/6.8 with a coupling constant (d, 7.5 Hz) assigned the ß-configuration of the glucose moiety, (Supplementary Material; Figures S16–S18). The obtained chemical shifts were verified using data from the literature [43].

#### 2.2.5. Nucleoside Compound

**Figure 2:**
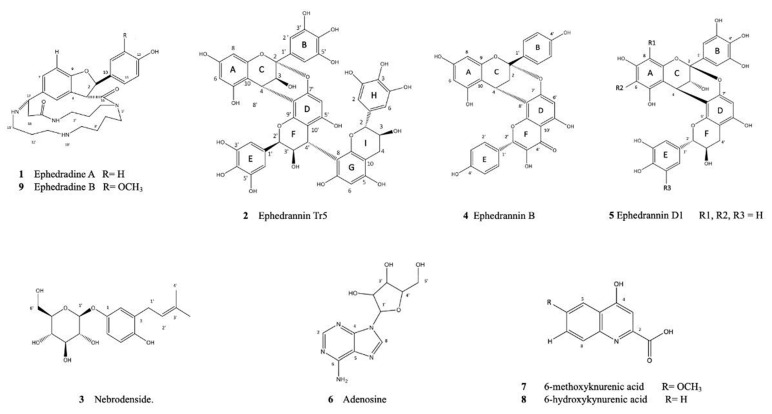
Structures of the isolated compounds **1**–**9**.

Compound **6** was obtained as a white powder with a negative ESI-MS/MS fragmentation pattern (*m*/*z* = rel. int.): 267.16; [M-H] is similar to adenosine (C_10_H_13_N_5_O_4_, 267.2) and exhibits an ion peak at *m*/*z* 265.17; [C_10_H_11_N_5_O_4_] was previously isolated from *Ephedra herba* root [44] (Supplementary Material; Figure S6). The ^1^H NMR spectrum included characteristic signals for H-2 and H-8 of the ribonucleoside moiety at 8.3, with a correlation to C2 and C8 at 152.4 and 132.0°, respectively. Furthermore, the HSQC spectra indicate that the ß-anomeric proton of the ribose moiety resonates at 6.2, correlating to 96.0 of C1, and the olefinic alkene signal at 4.07 is related to 66.1 of C5′. All of the isolated compounds are compiled in Figure 2, (Supplementary Material; Figures S25–S27).

### 2.3. Cytotoxicity of Isolated Compounds

The isolated compounds (**1**–**9**) were evaluated for cytotoxicity against MCF7 cell lines to determine the IC_50_ and IC_90_ (Table 4) values. The percentage of viable cells decreased significantly for all isolated compounds in a dose-dependent manner. Quinoline compounds play a significant role in anticancer drug development, as they possess different mechanisms of action such as apoptosis, inhibition of angiogenesis, growth inhibition by cell cycle arrest, and disruption of cell migration [45,46]. Compound **7** (6-methoxy kynurenic acid; 6-mKYNA) showed the most potent cytotoxic effect against the MCF7 cell line with inhibitory concentrations of 86.9 and 134.9 µg/mL at IC_50_ and IC_90_, respectively.

As presented in Table 5, incubation of 6-mKYNA (30 and 60 µg/µL) with MCF7 cells showed a significant decrease in the levels of CA-19.9 and CA-125 in MCF-7 cells compared to untreated cells at 24, 48, and 72 h. The depletion of CA19-9 and CA-125 in MCF-7 cells was dose- and time-dependent. Indeed, 6-mKYNA at 30 and 60 µg/µL at 72 h was more efficient than at 24 and 48 h in improving the levels of CA-19.9 and CA-125. Notably, kynurenic acid (KYNA), the core structure of isolated 6-mKYNA, is an endogenous tryptophan metabolite produced endogenously in various types of peripheral cells, and exerts neuroprotective and anticonvulsant properties in the brain. It was previously reported that KYNA is present in the serum of cancer patients in concentrations ranging from 21.3 to 250 nM depending on the cancer type [45]. There is some doubt about the role of KYNA in cancer, suggesting a double-edged role in carcinogenesis and a lack of a clear mechanism [46]. In millimolar concentrations, KYNA exerts antiproliferative activity against several cancer cell lines [47].

Our results are in accordance with those of previous studies that indicated the cytotoxic activity of a similar compound, KYNA, which exerted antiproliferative activity against glioblastoma T98G cells (IC_50_ = 8.9 mM) [48], HT-29 colorectal (IC_50_ = 4.4 mM) [49], and Caki-2 renal cancer cells (IC_50_ = 2.1 mM) [50].

This study represents highlights evidence reporting the potential use of methoxy derivatives of KYNA in breast cancer. Moreover, the established cytotoxic activity reveals that the isolated methoxy analog of KYNA is more potent than its hydroxy derivative in breast cancer cell lines. To our knowledge, this is the first report to indicate the cytotoxicity of 6-methoxy kynurenic acid, which highlights its potential for designing a new cytotoxic drug candidate.

### 2.4. Molecular Modeling

#### 2.4.1. Molecular Docking

To predict the possible mechanism that accounts for the inhibitory activity on growth achieved by the quinoline-2-carboxylic acid derivative (6-mKYNA), a molecular docking study was performed. It is worth mentioning that several quinoline derivatives have been reported as promising inhibitors of the serine/threonine type; casein kinase 2 (CK2) [51,52,53] is one of the protein kinases that is involved in many cellular functions, such as proliferation and apoptosis, and has been revealed to be overexpressed in various human cancer cells [54]. CK2 is intimately related to the progression of MCF7 breast adenocarcinoma [55]. Consequently, the X-ray crystal structure of CK2 co-crystallized with pyrimido [4,5-c] quinoline-8-carboxylic acid [56] was downloaded from the PDB (http://www.rcsb.org/ (accessed on 1 December 2022)) (PDB code: 3r0t) and used in the current docking investigation. The docking protocol used the MOE 2016.08 molecular modeling program in this study.

**Figure 3:**
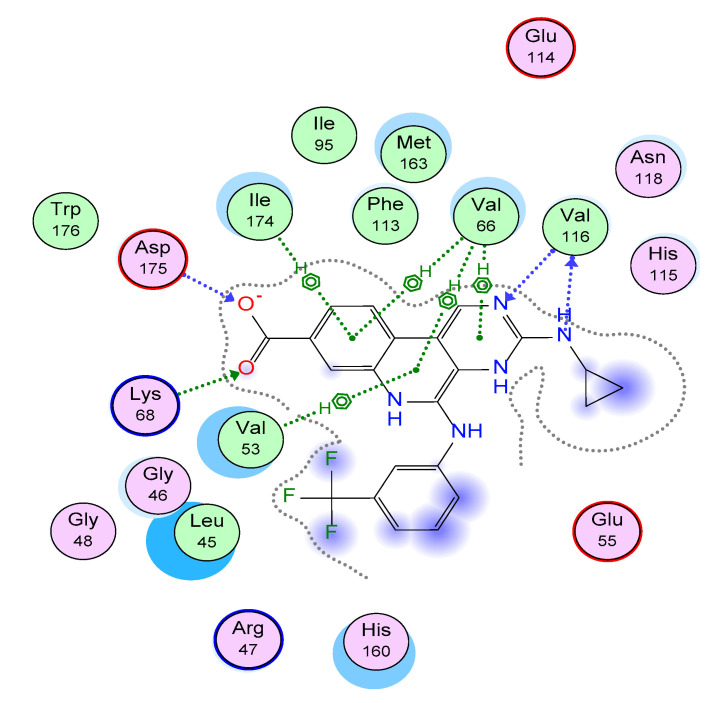

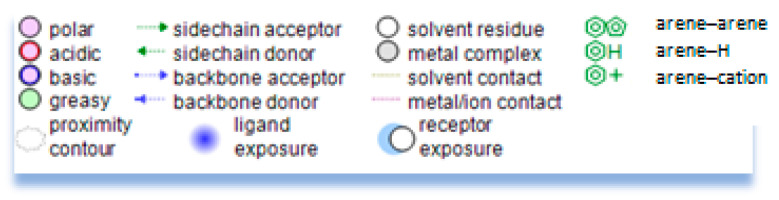
2D ligand interaction diagram of the re−docked pose in the CK2 binding site.

First, a validation step encompassing the redocking of the native ligand into the enzyme-binding site was completed. The validation parameter RMSD = 0.5939 A°, together with the comparable binding mode of the redocked pose to that of the co-crystallized ligand, were used to validate the adopted docking protocol. As shown in Figure 3, the binding mode of the redocked ligand includes three types of intermolecular interactions: H-bonding, ionic, and *pi*-H hydrophobic interactions. A deep look revealed the formation of two H-bonding interactions with the Val116 residue in the CK2 binding site through the N atom of the pyrimidine ring and the side chain NH of the native ligand. Another H-bonding interaction was observed between Asp175 and the Carbon atom of the carboxylic group. In addition, an ionic interaction was observed with the Lys68 amino acid via the O atom of the carboxylic group. Additionally, a network of *pi*-H hydrophobic interactions was formed with amino acids Val53, Val66, and Ile174 through the pyrimido [4,5-c] quinolone core of the native ligand [56].

**Figure 4:**
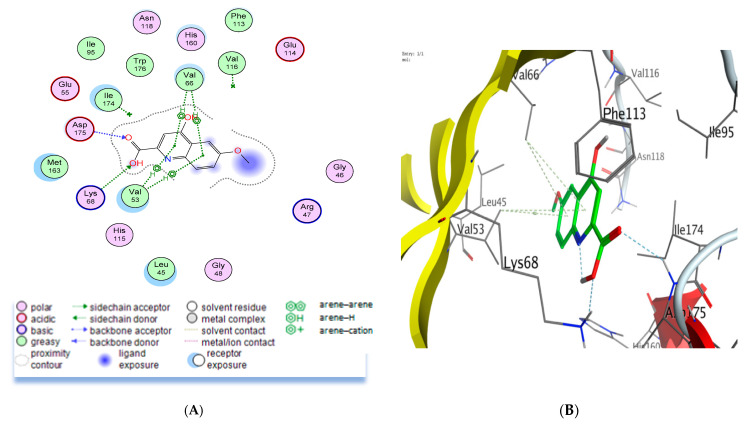
Ligand interaction diagram of 6-mKYNA (8) with the CK2 ATP binding site in 2D representation (**A**) and 3D representation (**B**).

Consequently, docking procedures were performed for the quinoline-2-carboxylic acids (6_mKYNA). The results demonstrate that our studied compound has a binding mode similar to that of the co-crystallized ligand, whereby 6-mKYNA reveals two types of intermolecular interactions with the essential residues in the binding site: H bonding and *pi*-H hydrophobic interactions (Figure 4). Two H-bonding interactions were observed with Lys68 and Asp175 essential amino acids in the CK2 binding site via the carboxylic OH and C=O groups of 6-mKYNA, respectively. In addition, *pi*-H hydrophobic interactions were displayed with Val53 and Val66 residues through the quinoline core. This binding pattern suggests that CK2 enzyme inhibition may be a possible mechanism for the observed anti-proliferative activity of 6-mKYNA.

#### 2.4.2. Pharmacokinetic and Drug-Likeness Aspects Prediction

**Figure 5:**
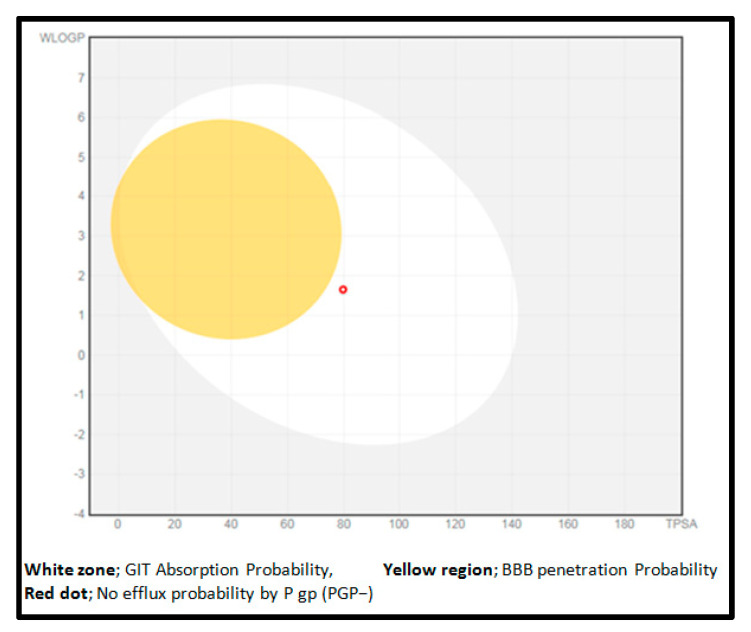
Human intestinal absorption (HIA) and blood−brain barrier (BBB) plots for all studied 6−mKYNA.

The Swiss ADME (http://www.swissadme.ch/ (accessed on 1 December 2022)) web server was used to predict the pharmacokinetics and drug-likeness features of the target compound “6-mKYNA.” Blood–brain barrier penetration (BBB), human gastrointestinal absorption (HIA), *p*-glycoprotein (P-gp) permeability, and interactions with cytochrome P450 isomers (CYP) were predicted. The results are shown as BOILED-Egg, which is a 2D plot presented via the calculated TPSA and logP of the checked compound (Figure 5) [57]. The results reveal that the studied compound (**7**) may be absorbed through the gastrointestinal tract, and it may not be a substrate for P-glycoprotein (PGP-), thus reducing the opportunity for its efflux by cancer cells [58]. Additionally, 6-mKYNA (7) is predicted to display non-inhibitory activities on cytochrome P450 isomers, and thus is expected to mediate no drug–drug interactions [59] (Table 6).

In addition to the compliance of 6-mKYNA to the Lipinski, Ghose, Egan, Veber, and Muegge rules, we also assessed its ability to be applied as an oral drug candidate. Lipinski et al. stated that oral bioavailability is probable if at least three of the following rules are followed: molecular weight ≤ 500 Da, number of hydrogen bond acceptors ≤ 10, number of hydrogen bond donors ≤ 5, and partition coefficient log P ≤ 5 [59].

The Ghose filter [60] defines drug-likeness boundaries as follows: calculated log P is between “−0.4–5.6”, molar refractivity is between “40–130”, MW is between “160–480”, and the total number of atoms is between “20–70.” The Veber rule [61] outlines drug-likeness criteria as a rotatable bond count ≤ 10 and polar surface area (PSA) ≤ 140. Egan’s filter [62] comprises WLOGP (lipophilicity) ≤ 5.88 and a total polar surface area ≤ 131.6. Muegge’s [63] constraints include molecular weight from 200 to 600, total polar surface area ≤ 150, XLOGP3 (lipophilicity) from −2 to 5, number of rings ≤ 7, number of heteroatoms > 1, number of carbons > 4, number of rotatable bonds ≤ 15, hydrogen bond donors ≤ 5, and hydrogen bond acceptors ≤ 10. As displayed in Table 6, a good oral bioavailability score (0.56) is predicted for 6-mKYNA, which is in total agreement with all of the above-mentioned rules, revealing no violations.

## 3. Materials and Methods

### 3.1. General

NMR Instrument: ^1^H-NMR (400 MHz, MHZ), ^13^C-NMR (100 MHz, MHZ), and HSQC were measured on a Bruker high-performance digital FT-NMR spectrometer Avance III 400 MHZ. The NMR spectra were recorded in CDCl_3_ and DMSO-*d6*, and the MeOD chemical shifts were expressed in δ (ppm) relative to TMS as an internal standard. Mass spectrometry: the EI/MS spectra were obtained on ESI-MS/MS positive and negative ion acquisition modes that were carried out on a XEVO TQD triple quadruple instrument (Waters Corporation; Milford, MA, USA, mass), with column, ACQUITY UPLC-BEH C18 1.7 µm (2.1 × 50 mm), and the flow rate was 0.2 mL/min. The solvent system consisted of a gradient elution of (A) water containing 0.1% formic acid and (B) acetonitrile.

### 3.2. Plant Material

The plant samples were collected from the Egyptian market, and a voucher specimen was identified by a senior botanist researcher at the Flora and Taxonomy Research Department, Agricultural Museum, Giza, Egypt. The voucher specimen (No. 20210010) was deposited in the Herbarium of the Pharmacognosy Department, Faculty of Pharmacy, October 6 University.

### 3.3. Extraction and Fractionation

The ground aerial parts of the fresh plant material (500 g) were moistened with ammonia and extracted with dichloromethane (CH_2_Cl_2_) (3 × 2 L) at room temperature and evaporated at reduced pressure to yield a residue of 15 g. The latter was partitioned between n-hexane (3 × 1 L), CH_2_Cl_2_ (3 × 1 L), EtOAc (3 × 1 L), and methanol MeOH (3 × 1 L). The solvents were evaporated under vacuum to produce the crude n-hexane (0.15 g), CH_2_Cl_2_ (4.9 g), EtOAc (2.7 g), and MeOH (3.0 g) fractions. The four fractions were subjected to in vivo antitumor activity analysis to determine the most active compound.

### 3.4. Experimental Model and Cell Culture

The experiment was conducted using the guidelines developed by the Animal Care and Use Committee of the October 6 University (Code No: 20210902). Adult mice, weighing approximately 25 ± 2 g, were purchased from Cairo University, Faculty of Veterinary Medicine. They were housed in cages in an air-conditioned environment with a temperature of 22 °C, relative humidity of 60%, and a light period of 8:00 to 20:00. Each animal was fed a daily diet ad libitum during the acclimatization period. Cell viability was assessed by mitochondrial-dependent reduction of yellow MTT (3-(4,5-dimethylthiazol-2-yl)-2,5-diphenyl tetrazolium bromide) to purple formazan [64]. The cells were suspended in DMEM for MCF7, 1% antibiotic–antimycotic mixture (10,000 U/mL potassium penicillin, 10,000 µg/mL streptomycin sulfate, and 25 µg/mL amphotericin B), and 1% L-glutamine at 37 °C under 5% CO_2_. For ELISA analysis of the CA-19.9 and CA-125 tumor biomarkers, the stored frozen cells were removed from liquid nitrogen, rapidly thawed by gentle agitation of the vial in a 37 °C water bath, and then diluted by gently pipetting up and down using 3 mL of pre-warmed complete growth medium under serial conditions. The cell suspension was diluted with complete medium to a concentration of 5 × 10^4^ cells/mL. The cell suspension (100 µL) was pipetted into each well of 96-well plates (approximately 5 × 10^3^ cells/well). Sandwich antigen capture ELISA assays were recorded for CA19-9 (Catalog # ELH-CA19-9, Ray Biotech Life, Inc., Norcross, GA, USA) and CA CA-125 (Catalog Number SE120017, Sigma Aldrich, St. Louis, MO, USA) in the cell culture supernatant after 24, 48, and 72 h at 450 nm.

### 3.5. Ehrlich Ascites Carcinoma Test

The EAC cells were provided by the National Cancer Institute and were maintained in vivo in Swiss albino mice via intraperitoneal transplantation (2 × 10^6^ cells per mouse) into all groups except the control group (65). The animals were divided into seven groups consisting of ten animals, two control groups, and five treatment groups (groups III to VII), and the treatment started 21 days after Ehrlich ascites carcinoma implantation (Table 7). Mice from each group were dissected on day 52 from the start of implantation. Ascitic fluid was aspirated from the mice for EAC volume and weight detection according to the method described by Badr et al. (2011) [65]. Blood was collected, centrifuged, and plasma tumor marker bioassays, including cancer antigen 125 (CA-125) and cancer antigen 19-9 (CA-19-9), were measured using ELISA kits (Ray Biotech Life, Inc., Norcross, GA, USA).

### 3.6. Isolation of Compounds from Bioactive Fraction

The EtOAc fraction was the most bioactive antitumor fraction; it was subsequently purified by fractionation using an Amberlite resin XAD18 column (2 × 50 cm) and a 75% MeOH mobile phase. The fractionation yielded nine subfractions (I → IX), and TLC screening showed a positive result with Dragendorff’s reagent for fractions I–III, where FI was 0.2 g, FII was 0.05 g, and FIII was 0.03 g.

FI was purified via Sephadex column chromatography LH-20 (2 × 30 cm) using 100% MeOH, and subfractions 1–20 were subsequently fractionated on a silica gel column (2 × 30 cm) (CH_2_Cl_2_/MeOH, gradient elution). From the silica column, only one subfraction showed a positive result with Dragendorff’s sprayer (f14-17, 79 mg), which was purified using a flash column (silica gel, using CH_2_Cl_2_, EtOAc, and MeOH) to yield subfractions FIa, FIb, and FIc.

FIa was purified via preparative TLC using (CH_2_Cl_2_:EtOAc:MeOH:H_2_O:NH_3_ 3:5:2:0.5:1) to afford compound **1** (10 mg). Compound **2** (15 mg), compound **3** (11 mg), and compound **4** (15 mg) were isolated from FIb after fractionation by silica column using a gradient elution of CH_2_Cl_2_, EtOAc, C_3_H_6_O, and MeOH. Compound **5** (10 mg) was purified from FIc via crystallization.

Compound **6** (11 mg), compound **7** (23 mg), and compound **8** (12 mg) were obtained after silica column chromatography (1 × 15 cm) of the FII fraction (CH_2_Cl_2_/MeOH 1:1) and further purified by Sephadex LH-20 (100% MeOH). Compound **9** (16 mg) was isolated from FIII after purification on a silica column (1 × 10 cm) using a gradient elution of CH_2_Cl_2_, EtOAc, C_3_H_6_O, and MeOH.

### 3.7. Cell Viability Assay MTT

Cells were seeded in 96-well plates (200 μL, 2 × 10^3^ cells/well) and grown for 24 h at 37 °C under hypoxic conditions (5% CO_2_). Subsequently, the cells were treated with different concentrations of the compounds (100, 50, 25, 12.50, 6.25, 3.13, 1.56, and 0.78 μM). After 72 h of incubation, the medium was aspirated, 40 uL of MTT salt (2.5 μg/mL) was added to each well and incubated for a further four hours at 37 °C under 5% CO_2_. To stop the reaction and dissolve the crystals formed, 200 μL of 10% sodium dodecyl sulfate (SDS) in deionized water was added to each well and incubated overnight at 37 °C. The positive control, doxorubicin, at a concentration of 100 µg/mL, was used as a known cytotoxic natural agent with 100% lethality under the same conditions. The absorbance was measured at a wavelength of 595 nm and a reference wavelength of 620 nm using a spectrophotometric ELISA plate reader (SpectraMax^®^ i3, Molecular Devices, Nova Biotech, CA, USA). DMSO was the vehicle used for the dissolution of plant extracts, and its final concentration in the cells was less than 0.2%. (18–20). The percentage change in viability was calculated according to the following formula: ((reading of extract/reading of negative control) − 1) × 100. Probity analysis was carried out to determine the IC_50_ and IC_90_ using the SPSS 11 program.

### 3.8. Sandwich Antigen Capture ELISA Assays Recorded for CA-19.9 and CA-125

MCF-7 cells were seeded in ELISA plates (6-well plates) as control cells and cells with different concentrations of 6-methoxy kynurenic acid (30 and 50 µg/µL), and incubated at 37 °C, 95% humidity, and 5% CO_2_. Three plates were prepared for each sample of treated and untreated cells as follows: plates for untreated cells (control plate), plates for treatment with 30 µg/µL 6-mKYNA, and plates for treatment with 60 µg/µL 6-mKYNA. After 24 h, the media were removed from the plates and replaced with 10 mL of fresh media for the control plate, while the treatment plates received 6-methoxy kynurenic acid (30 and 60 µg/µL). The plates were incubated at 37 °C, 5% humidity, and 5% CO_2_ for 72 h. The treated and control samples were taken at given time points and stored at −80 °C until the next step. Sandwich antigen capture ELISA assays were performed for CA-19.9 and CA-125 in the cell culture supernatant after 24, 48, and 72 h at 450 nm. The test was repeated three times, and the results were averaged.

### 3.9. Statistical Analysis

Statistical significance between samples and negative controls was tested using an independent *t*-test with the SPSS 11 program.

### 3.10. Molecular Modeling

#### 3.10.1. Molecular Docking Study

The Molecular Operating Environment (MOE 2016.08) program was used for the molecular docking studies. All of the minimizations were completed with MOE until the RMSD gradient of 0.05 Kcal.mol^−1^ Å^−1^ with the MMFF94 force field was reached. Automatic calculations of partial charges were performed. The triangle matching placement method and London dG scoring function were used as the docking protocols. The X-ray crystal structure of the CK2 enzyme co-crystallized with pyrimido [4,5-c] quinoline-8-carboxylic acid (PDB code: 3r0t) was downloaded from the Protein Data Bank in PDB format (http://www.rcsb.org/ (accessed on 1 December 2022)), and prepared as follows: (i) removal of the water molecules and ligands that are not included in the binding; (ii) protonate the 3D protocol with default parameters in the MOE. The studied compound was constructed in a 3D format via MOE. Then, it was exposed to the following: (i) 3D protonation of the structure; (ii) conformational analysis via systemic search; (iii) the conformer with the least energy was selected; (iv) the same docking protocol was applied to the original ligand [66].

#### 3.10.2. Pharmacokinetic and Drug-Likeness Aspects Prediction

ACD Labs Chemsketch version 11.01 was used to generate the chemical structure and SMILES notation of the checked compound. Then, the SMILES notation was nourished into the Swiss ADME (http://www.swissadme.ch/ (accessed on 1 December 2022) web server to predict the pharmacokinetic and drug-likeness characteristics.

## 4. Conclusions

Natural products are one of the most significant sources for drug development. This study demonstrated promising anticancer activity of the EtOAc fraction of *E. foeminea*. Nine metabolites were isolated, for the first time that belong to the alkaloid (quinoline and macrocyclic spermine), proanthocyanidin, nebrodenside, and adenosine classes. The results revealed that the isolated 6-methoxykynurenic acid (6-mKYNA) could be a promising new agent for the treatment of breast cancer, and that 6-mKYNA can directly inhibit CA-19.9 and CA-125 tumor biomarkers.

An in silico study was used to predict the possible anti-proliferative mechanism, and the results indicated a similarity between the binding mode of 6-mKYNA to the native ligand pyrimido [4,5-c] quinoline-8-carboxylic acid and the CK2 enzyme binding site. 6-mKYNA may also possess good ADMET properties, which indicates promising pharmacodynamic, pharmacokinetic, and physiochemical properties. According to the described findings, the evaluated compound is a prospective drug-like candidate that could be further developed into an active commercial anticancer drug.

### 4.1. Study Limitations

- The findings of this study have to be seen in light of some limitations in methodology. Despite the remarkable advantages of NMR spectroscopy, certain limitations exist, including the high cost and low sensitivity of NMR instruments to sufficiently sample concentrations, higher molecular weight molecules, and molecules with ionic states, which could sometimes lead to poor spectra that need to be addressed by researchers to enable future breakthroughs.
- These weak points for NMR could be overcome by the use of mass-spectroscopy analysis (ESI-MS), which was what the authors carried out in this study, where MS-based metabolomics provides a great technique for large molecular weight ionic compounds, as it provides a combination of sensitivity and selectivity platforms for such metabolomics research. Furthermore, other MS methodologies, such as different ionization procedures and mass analyzer technologies, can be employed to improve the number of detectable metabolites.

### 4.2. Prospective Studies

- Further in vitro and in vivo studies are required to fully explore the possible biological activities of the non-alkaloid constituents of *E. foeminea*.
- Structural modifications to improve constituent activities, safety, and pharmacokinetics can be used as templates for the design of new biologically active molecules from *Ephedra* species.
- Endophytic fungal strains associated with *Ephedra* species are potential sources for development in chemical, microbiological, and pharmacological fields.
- To the best of our knowledge, this is the first study to demonstrate the cytotoxicity of 6-methoxy kynurenic acid, highlighting its potential utility in the development of a novel cytotoxic drug candidate.
- This study could be an incentive to develop and expand research on the semi-synthesis of new C-6 substituted kynurenic acid derivatives that could be used as herbal dietary supplements for cancer patients after proper clinical trials.

## Figures and Tables

**Table 1:** Effects of *Ephedra* fractions and 5-fluorouracil (5FU) on tumor volume (ml) and weight (g) after Ehrlich ascites carcinoma (EAC) implantation.

		Tumor Volume	Tumor Weight
I	Normal control A	0.0 ± 0.0 ^a^	0.0 ± 0.00 ^a^
II	EAC control	2.80 ± 0.13 ^e^	1.70 ± 0.07 ^e^
III	EAC + Hexane fraction	2.08 ± 0.20 ^d^	1.25 ± 0.05 ^d^
IV	EAC + Dichloromethane fraction	2.42 ± 0.07 ^d^	1.37 ± 0.06 ^d^
V	EAC + Ethyl acetate fraction	1.76 ± 0.06 ^c^	0.90 ± 0.03 ^c^
VI	EAC + Methanol fraction	1.90 ± 0.03 ^c^	1.13 ± 0.07 ^c^
VII	EAC + 5FU (20 mg/kg)	1.21 ± 0.08 ^b^	0.68 ± 0.05 ^b^

Values are given as mean ± SD; control group (I) did not receive any treatment; EAC control (II) received Ehrlich ascites cells (2 × 10^6^ cells per mice) via intraperitoneal transplantation; group data followed by the same superscripted letter are not significantly different from each other at *p* ≤ 0.05.

**Table 2:** ^1^H and ^13^C (400 MHz) data of compounds **1**, **3** (CDCl_3_), **6** (DMSO) **7**, **8**, and **9** (CD_3_OD) (δ in ppm).

No.	1		3		6		7		8		9	
	^13^C	^1^H	^13^C	^1^H	^13^C	^1^H	^13^C	^1^H	^13^C	^1^H	^13^C	^1^H
1			157.3									
2	113.1	4.95 (d, 11.5)	95.9	7.26 (s)	152.4	8.36 (1s)	150.0		145.0		105.2	6.62 (1d, 12.1)
3	59.5	4.10 (d, 11.6)	128.1				110.0	7.03 (s)	104.0	5.94 (s)	49.9	4.70 (1d, 12.2)
4	127.2		153.2		149.0		165.0		164.2		126.2	
5	122.5	7.20 (s)	96.2	7.23 (d, 8.4)	119.3		115.3	6.90 (s)	110.5	7.45 (s)	127.6	7.52 (1s)
6	148.2		78.8	7.21 (d, 8.7, 2.4)	156.2	7.82-NH (brs)	158.2		153.7		131.9	
7	122.2	7.10 (d, 8.2)					126.5	7.41 (d, 9.0)	110.8	7.43 (d, 9.3)	126.8	8.20 (1d, 8.0)
8	114.5	6.61 (d, 8.0)			132.0	7.62 (s)	130.0	8.01 (d, 10)	127.6	7.39 (d, 9.7)	126.4	7.15 (d, 7.8)
9	150.3						143.2		142.0		147.5	
10	149.2						115.0		120.0		137.0	
11	122.4	7.11 (d, 9.3)						3.81, 3H, br s, OMe			108.2	6.64 (brs)
12	119.2	7.10 (d, 9.4)					174.6		168.3		149.1	
13	150.1	4.95 (d, 11.5)									148.2	
14	114.5	6.61 (d, 8.5)									128.0	6.81 (d, 8.3)
15	122.4	7.11 (d, 8.4)									105.7	6.72 (d, 8.1)
16	169.1										177.5	
17	64.7	4.01, 3.91–3.96 (m)									70.0	4.21–4.35 (m)
18	38.6	2.35, 2.54 (d, 13.2)									34.7	2.95, 2.70 (d, 12.9)
19	169.1										178.0	
1′		8.11 (t, 11.4)	29.7	2.40 (d, 7.3)	96.0	6.20 (d, 6)						7.50 (t)- NH
2′	70.6	3.62–3.66 (m)	127.2	5.95 (t, 6.0 Hz)	72.5	3.60 (dd, 4, 5)					65.5	3.61–3.62 (m)
3′	25.8	1.60–1.62 (m)	130.0		71.0	3.40 (dd, 3, 4.6)					20.3	1.72–1.74 (m)
4′	71.9	3.40–3.44 (m)	15.0	1.60 (s)	85.9	3.96 (dd, 3, 3.6)					48.9	3.20–3.23 (m)
5′			25.4	1.60 (s)		4.07 (dd, 4, 12)						
6′	72.5	3.53–3.55 (m)									71.5	3.55–3.56 (m)
7′	32.6	1.52–1.54 (m)									27.2	1.20–1.22 m)
8′	30.3	1.31–1.33 (m)									26.2	1.07–1.08 (m)
9′	39.1	2.13–2.15 (m)									50.2	2.91–2.93 (m)
10′		1.7 1–1.73 (m)										1.50–1.52 (m)
11′	39.6	2.11–2.12 (m)									49.6	2.84–2.86 (m)
12’	27.5	1.60–1.62 (m)									22.7	1.72–1.74 (m)
13′	39.2	2.33–2.35 (m)									49.5	2.70–2.72 (m)
14′		2.17–2.19 (m)										
1″			85.8	6.83 (d, 7.5 Hz)								
2″			70.4	4.20–4.22 (m)								
3″			76.1	3.45–3.46 (m)								
4″			71.5	3.60–3.62 (m)								
5″			72.5	3.45–3.46 (m)								
6″			62.5	3.70, 3.62 (dd, 11.7, 5.1)								

**Table 3:** ^1^H and ^13^C-NMR (400 MHz) data for isolated proanthocyanidin compounds (**2**, **4**, and **5**) in CDCl_3_ (δ in ppm).

		2		4		5	
		^13^C	^1^H	^13^C	^1^H	^13^C	^1^H
**C**	2	114.0		113.7		112.0	
	3	67.1	5.24 (d, 3.4)	35.3	2.35, 2.49 (d, 4.12)	77.7	7.13 (d, 3.5)
	4	56.2	3.9 (d, 3.4)	67.5	3.90 (t)	36.5	2.65 (d, 3.5)
**A**	5	167.7		161.3		154.0	
	6	130.1	5.23 (brs)	79.7	4.08 (brs)	127.0	5.25 (brs)
	7	167.7		152.1		154.1	
	8	130.1	5.23 (brs)	78.8	6.72 (brs)	130.2	5.32 (brs)
	9	158.9		157.2		160.1	
	10	105.0		102.3		100.4	
**B**	1′	130.8		127.3		132.4	
	2′	130.0	7.61 (s)	138.2	6.60 (d, 8.5)	129.2	7.64 (s)
	3′	144.2		114.9	6.42 (d, 8.5)	140.2	
	4′	129.7		153.4		130.8	
	5′	144.0		114.9	6.42 (d, 8.5)	147.3	
	6′	130.0	7.61 (s)	127.5	6.60 (d, 8.5)	129.2	7.62 (s)
**F**	2′	71.2	4.37 (d, 7.3)	150.2		67.5	4.25 (d, 7.4)
	3′	114.2	4.91–4.93 (m)	135.3		60.2	4.17–4.15 (m)
	4′	56.1	3.82 (d, 2.5)	179.1		34.2	2.21, 2.41 (d, 5.2, 14.3)
**D**	5′	105.0		162.1		151.9	
	6′	115.0	5.00 (s)	79.5	7.75 (s)	130.5	5.25 (s)
	7′	158.1		161.2		149.8	
	8′	103.4		107.2		128.0	
	9′	157.0		151.2		152.5	
	10′	105.4		105.2		103.2	
**E**	1′	130.8		123.1		130.1	
	2′	130.6	7.62 (brs)	130.5	6.63 (d, 8.2)	130.6	7.4 (brs)
	3′	144.0		115.8	6.65 (d, 8.4)	142.3	
	4′	129.7		155.2		144.5	
	5′	144.0		114.7	6.41 (d, 8.4)	112.3	6.7 (d, 8.1)
	6′	130.0	7.62 (brs)	138.2	7.67 (d, 8.2)	130.1	7.3 (d, 8.1)
**I**	2″	71.2	4.37 (d, 9.4)				
	3″	68.1	4.15–4.17 (m)				
	4″	37.9	2.25, 1.80 (d, 8.5, 16.3)				
**G**	5″	167.7					
	6″	130.1	5.24 (s)				
	7″	167.7					
	8″	105.0					
	9″	157.0					
	10″	103.5					
**H**	1	128.8					
	2	131.2	7.43 (brs)				
	3	144.0					
	4	129.7					
	5	144.0					
	6	131.1	6.20 (brs)				

**Table 4:** IC_50_ and IC_90_ results of the studied compounds against MCF-7 cells.

Sample Code	Name	Class	IC_50 (_µM)	IC_90 (_µM)	Remarks
Compound **1**	Ephedradine A	Macrocyclic spermine bjtalkaloids	- *	- *	43.5% at 100 mM
Compound **2**	Ephedrannin Tr5	Proanthocyanidins	- *	- *	24.9% at 100 mM
Compound **3**	Nebradenside	Phenol	- *	- *	27.2% at 100 mM
Compound **4**	Ephedrannin B	Proanthocyanidins	- *	- *	27.6% at 100 mM
Compound **5**	Ephedrannin D1		- *	- *	25.3% at 100 mM
Compound **6**	adenosine	Nucleoside	- *	- *	15.7% at 100 mM
Compound **7**	6-Methoxy knurenic acid	Quinoline alkaloids	86.9	134.9	58.1% at 100 mM
Compound **8**	6-Hydroxy kynurenic acid		- *	- *	31.2% at 100 mM
Compound **9**	Ephedradine B	Macrocyclic spermine alkaloids	- *	- *	32.2% at 100 mM
Doxorubicin			45.2	- *	100% at 100 mM
DMSO			- *	- *	3% at 100 mM
Negative control			- *	- *	0%

*—IC_50_ more than 100 mM.

**Table 5:** Effects of different concentrations of 6-methoxy kynurenic acid (30 and 60 µg/µL) on the levels of CA-19.9 and CA-125 in MCF-7 cell supernatants.

Groups	Treatment Description	CA-19.9 (ng/mL)			CA-125 (ng/mL)		
		24 h	48 h	72 h	24 h	48 h	72 h
I	Control	6.54 ± 0.68 ^cA^	6.69 ± 0.76 ^cA^	6.66 ± 0.51 ^cA^	3.12 ± 0.28 ^cA^	3.18 ± 0.11 ^cA^	3.32 ± 0.10 ^cA^
II	30 µg/µL	4.50 ± 0.36 ^bC^	3.50 ± 0.49 ^bB^	3.10 ± 0.61 ^bA^	2.39 ± 0.16 ^bC^	1.73 ± 0.14 ^bB^	1.26 ± 0.10 ^bA^
III	60 µg/µL	3.54 ± 0.26 ^aC^	2.63 ± 0.10 ^aB^	2.18 ± 0.32 ^aA^	1.96 ± 0.20 ^aC^	1.04 ± 0.08 ^aB^	0.98 ± 0.12 ^aA^

Data are shown as the mean ± standard deviation. The control group did not receive any treatment; group data followed by the same superscript letter are not significantly different from each other at *p* ≤ 0.05. Small letters indicate significant differences at *p* ≤ 0.05 in the same column and capital letters indicate significant differences at *p* ≤ 0.05 in the same row.

**Table 6:** Computer-aided ADME screening of 6-mKYNA.

Cpd.	Pharmaco-Kinetics							Drug Likeness (# Number of Violations)					
	GIT Absorption	BBB Permeation	Pg-p Substrate	CYP2D6	CYP2C19	CYP1A2	CYP3A4	Lipiniski	Ghose	Veber	Egan	Muegge	Bioavailability Score
8	High	No	No	No	No	No	No	0	0	0	0	0	0.56

**Table 7:** Experimental Ehrlich ascites carcinoma-bearing model.

Code	Groups	Model
I	Normal control A	3 mL of distilled water, orally for 30 days
II	EAC control	Subcutaneous injection of 2 × 10^6^ cells/mice in water
III	EAC + Hexane fraction	30 mg/kg in water for 30 days in a single oral daily dose
IV	EAC + CH_2_Cl_2_ fraction	
V	EAC + EtOAc fraction	
VI	EAC + MeOH fraction	
VII	EAC + 5FU	20 mg/kg 5FU on alternate days for 30 days in a single daily dose (I.P) [25]

## Data Availability

The data presented in this study are available in this article.

## Supplementary Materials

The following supporting information can be downloaded at: https://www.mdpi.com/article/10.3390/molecules29010199/s1.
