# Supplementary material for: Bio-Guided Assay of Ephedra foeminea Forssk Extracts and Anticancer Activities: In Vivo, In Vitro, and In Silico Evaluations
Source: Molecules. 2023 Dec 29;29(1):199. doi: 10.3390/molecules29010199 (PMC10780881; doi:10.3390/molecules29010199)
Supplement: Supplementary file 1 [file molecules-29-00199-s001.zip › molecules-2758316-supplementary.pdf]

Supplementary Material

Bio-guided assay of *Ephedra foeminea* Forssk extracts and Anticancer Activities: *In vivo*, *In vitro*, and In Silico Evaluations

[1]Pharmacognosy Department, Faculty of Pharmacy, October 6 University, 6th of October City, Giza 12585, Egypt.
[2]Biochemistry Department, Faculty of Pharmacy, October 6 University, 6th of October City 12585, Egypt.
[3]Pharmaceutical Chemistry Department, Faculty of Pharmacy, October 6 University, Cairo, Egypt
* Correspondence: author: hebaelgizawy@o6u.edu.eg (H.A.E.G.)

Abstract: Bio-guided fractionation of *Ephedra foeminea* extract and *in vivo* antitumor biological evaluation revealed that the ethyl acetate (EtOAc) fraction was the most bioactive fraction. The phytochemical study of the most bioactive fraction (EtOAc) afforded the isolation of nine compounds for the first time from this species. Macrocyclic spermine alkaloids (1,9), proanthocyanidins (2,4,5), quinoline alkaloids (7,8), phenolic (3), and nucleoside (6) were identified and elucidated by spectroscopic analyses including 1D and 2D NMR, ESI-MS-MS spectrometry. The tested compounds showed moderate anticancer activity, except for the kynurenic acid derivative (6-mKYNA) which showed significant cytotoxicity and remarkable inhibition of CA-19.9 and CA-125 tumor biomarkers. An in-silico study was conducted to determine the anti-proliferative mechanism of 6-mKYNA by using the CK2 enzyme active site. Moreover, the ADME computational study suggested that 6-mKYNA is an effective candidate with a promising pharmacokinetic profile and therapeutic potential against various wide array of cancer diseases.

Keywords: *Ephedra foeminea*; alkaloids; proanthocyanidins; 6-methoxy kynurenic acid; tumor marker; in silico; ADME

Fragmentation pattern of nine isolated compounds

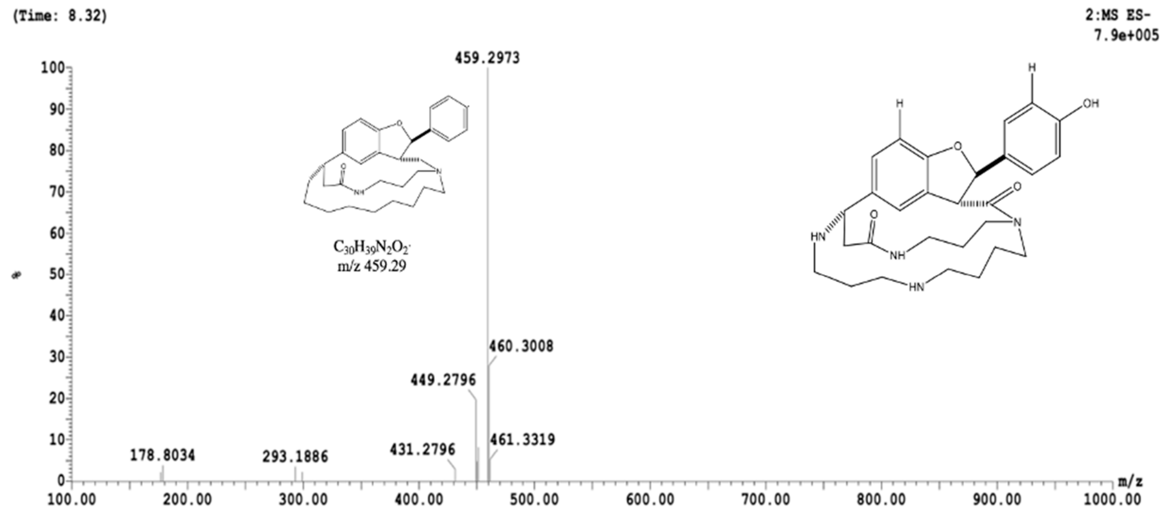

Figure S1. ESI-MS-MS spectral of compound 1 (Ephedradine A).

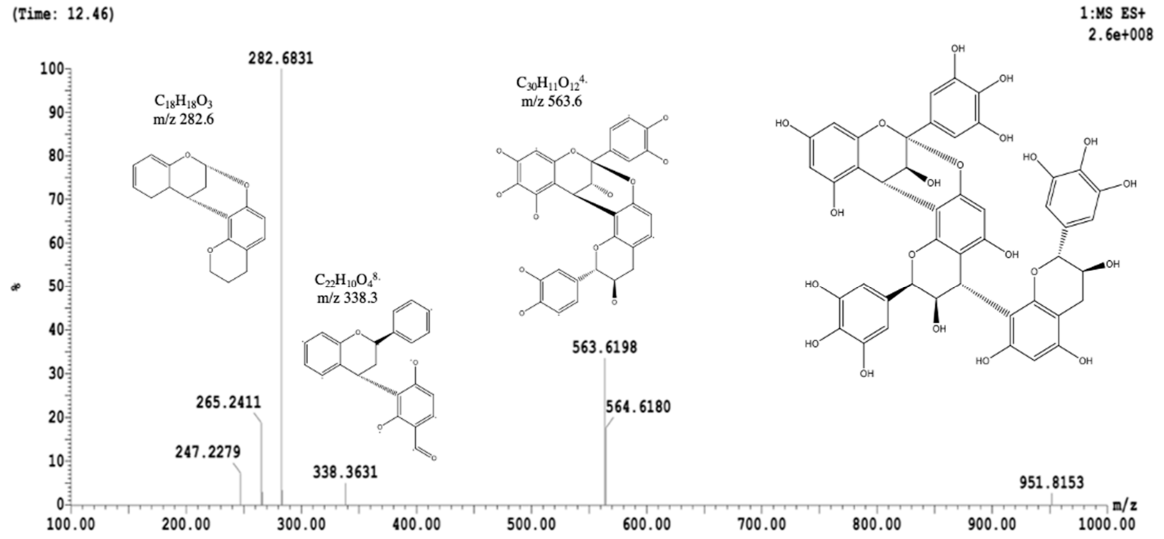

Figure S2. ESI-MS-MS spectral of compound 2 (Ephedrannin Tr5).

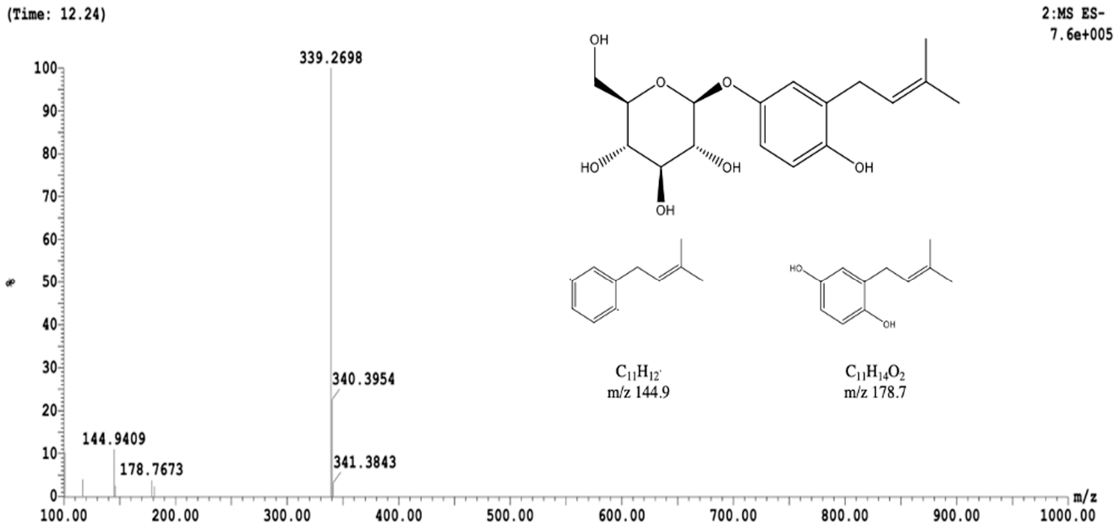

Figure S3. ESI-MS-MS spectral of compound 3 (Nebrodenside).

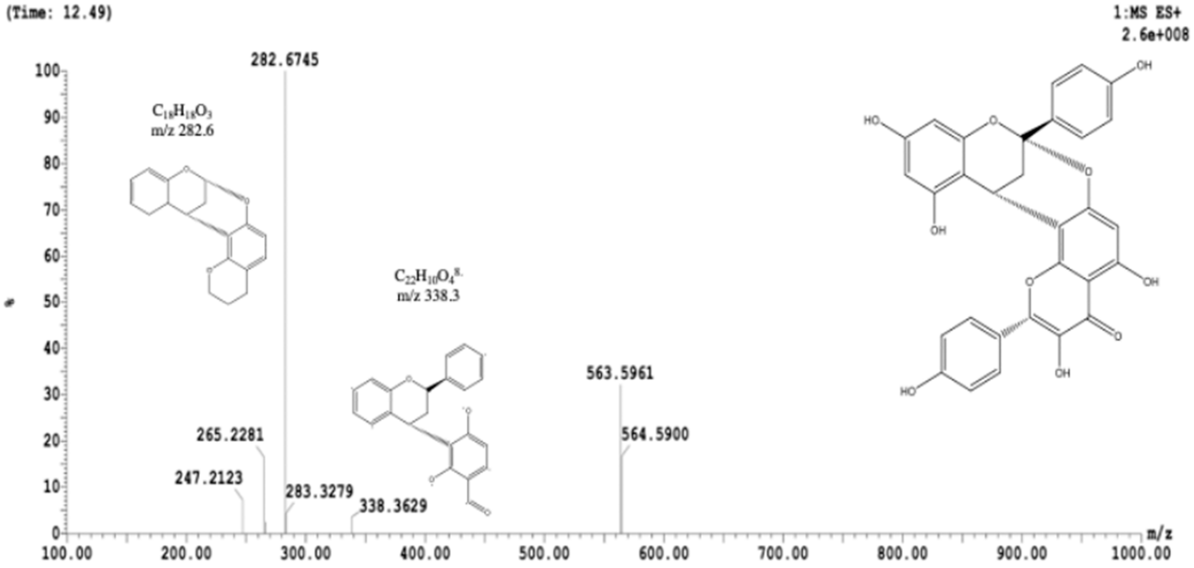

Figure S4. ESI-MS-MS spectral of compound 4 (Ephedranin B).

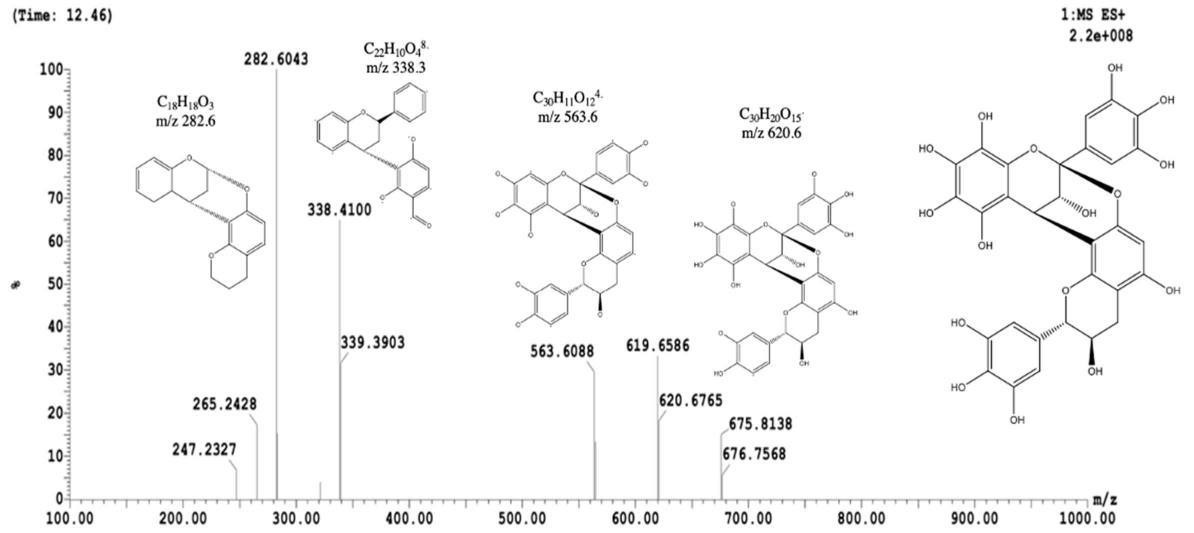

Figure S5. ESI-MS-MS spectral of compound 5 (Ephedranin D1).

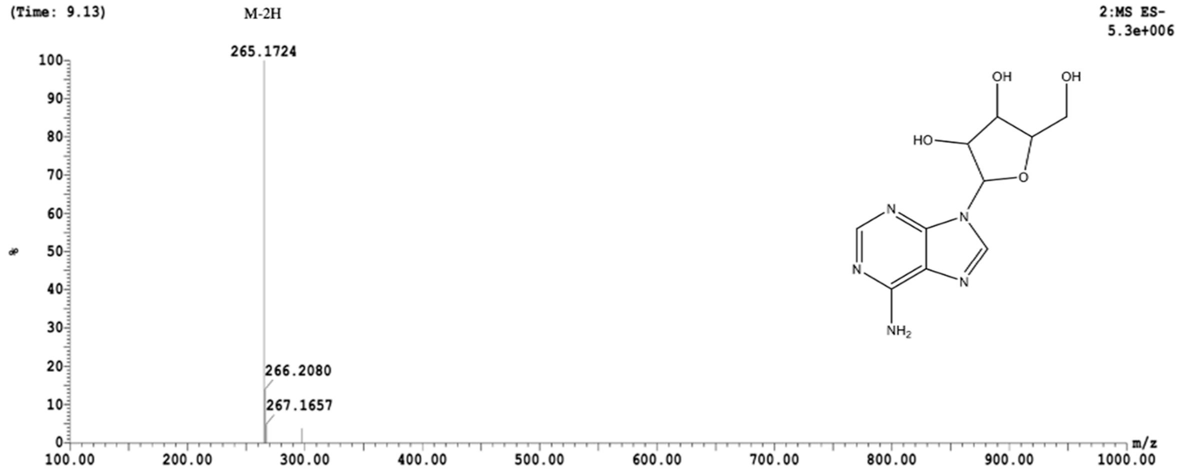

Figure S6. ESI-MS-MS spectral of compound 6 (Adenosine).

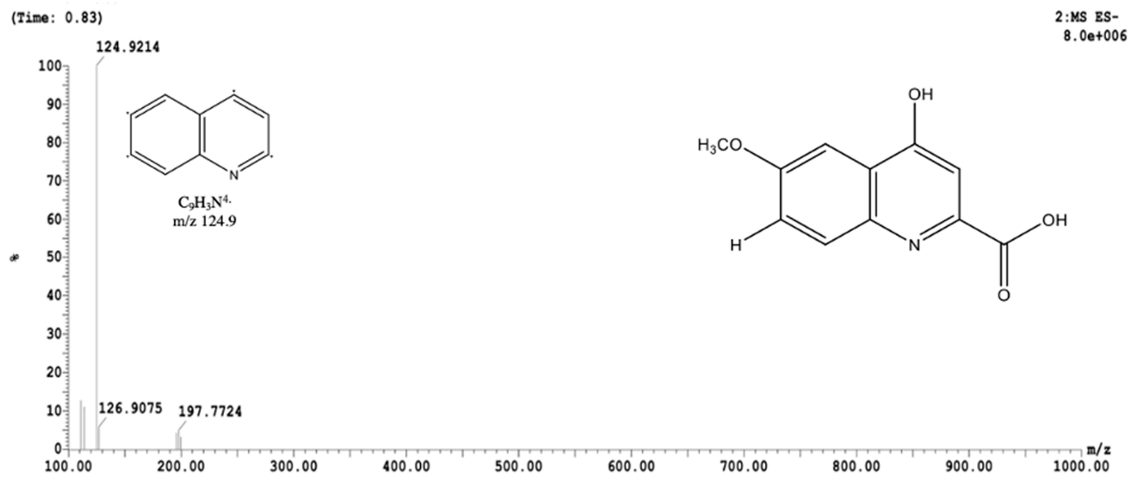

Figure S7. ESI-MS-MS spectral of compound 7 (6- methoxyknurenic acid).

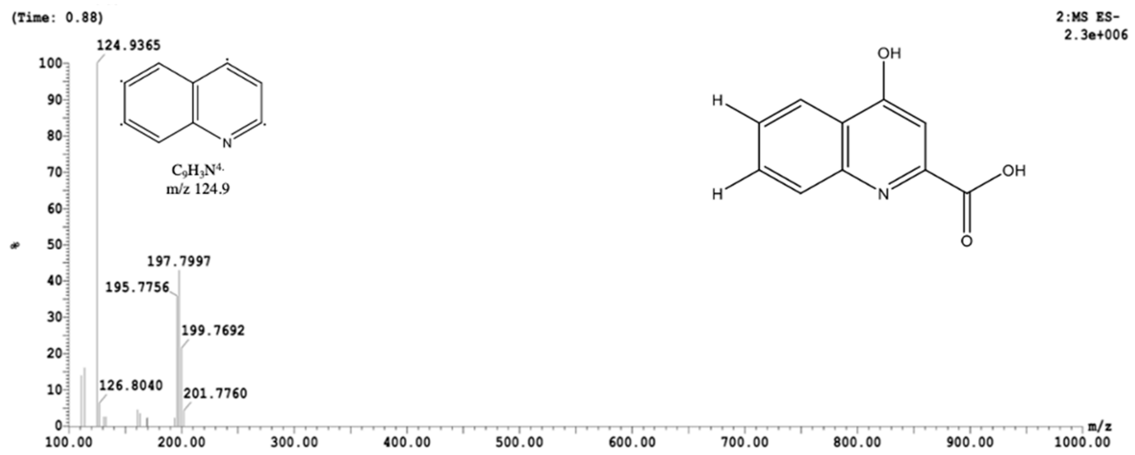

Figure S8. ESI-MS-MS spectral of compound 8 (6- hydroxyknurenic acid).

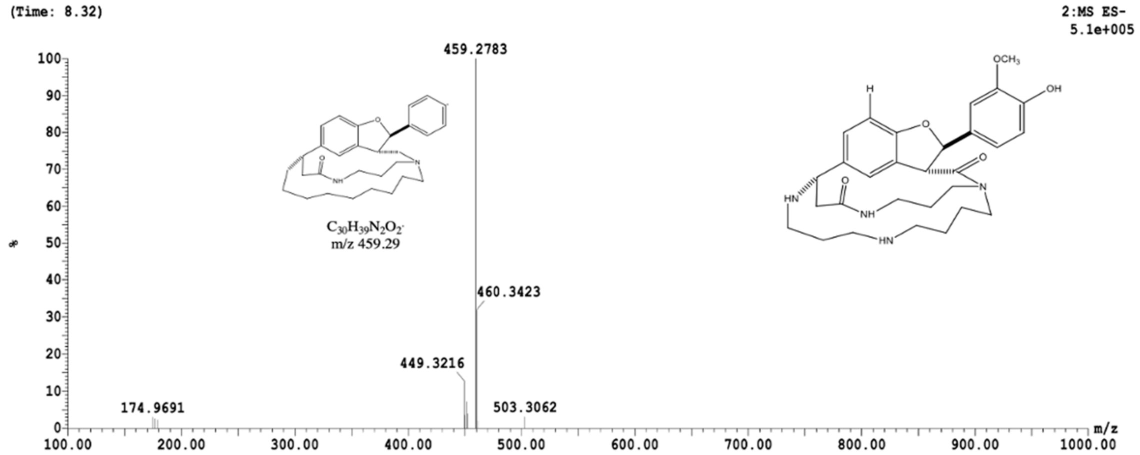

Figure S9. ESI-MS-MS spectral of compound 9 (Ephedradine B).

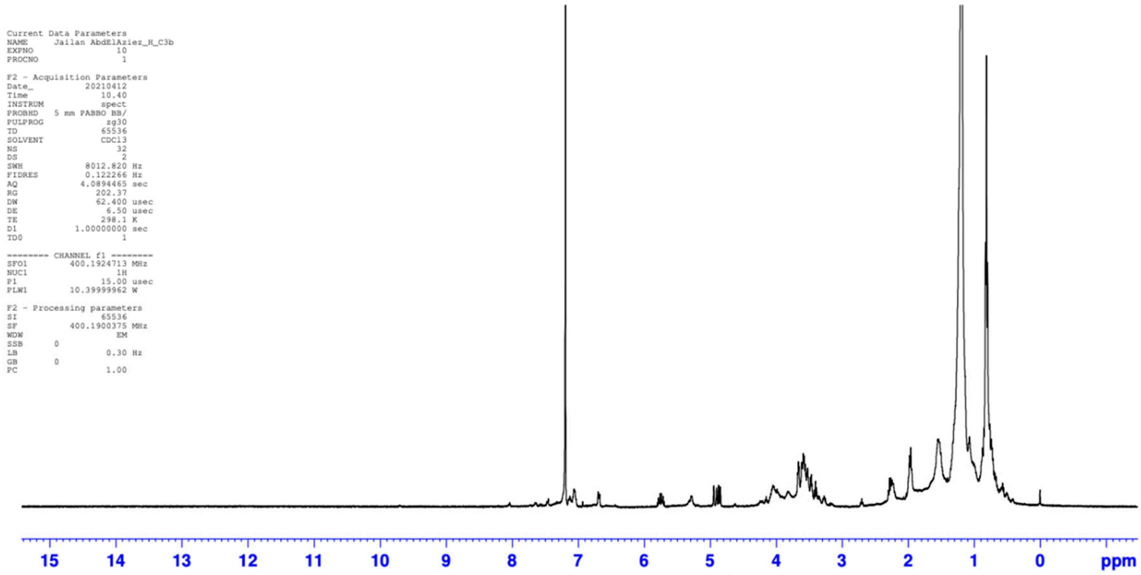

Figure S10: ^{1}HNMR Spectra of compound 1 (Ephedradine A).

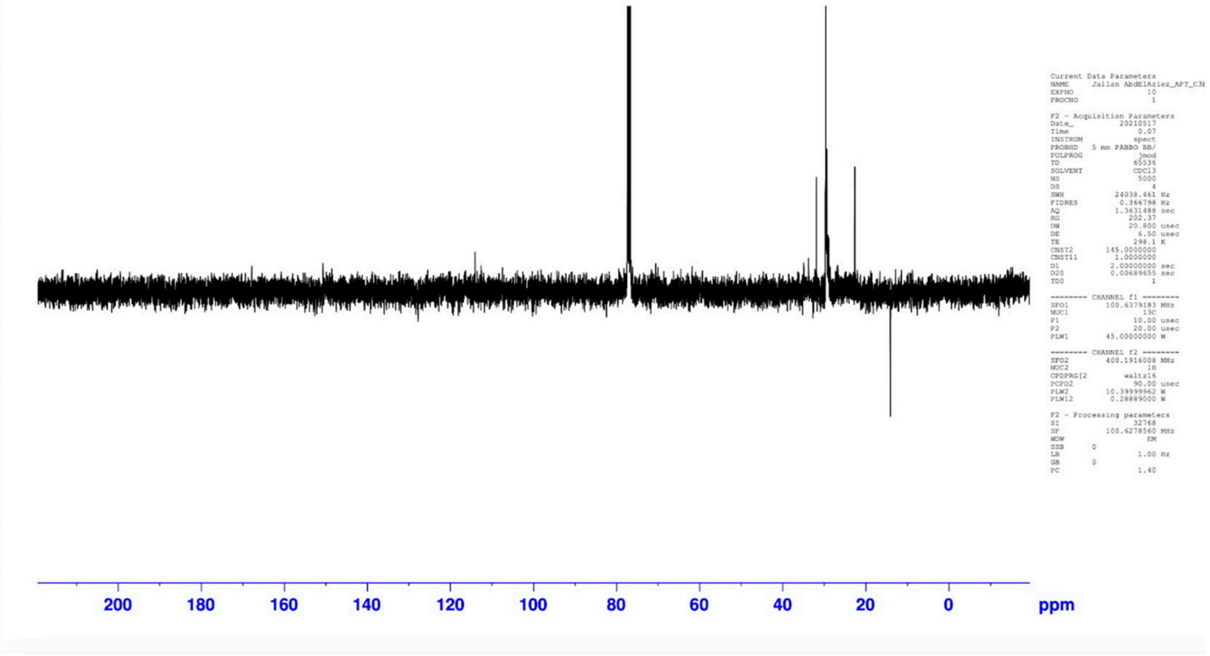

Figure S11: ^{13}C NMR Spectra of compound 1 (Ephedradine A).

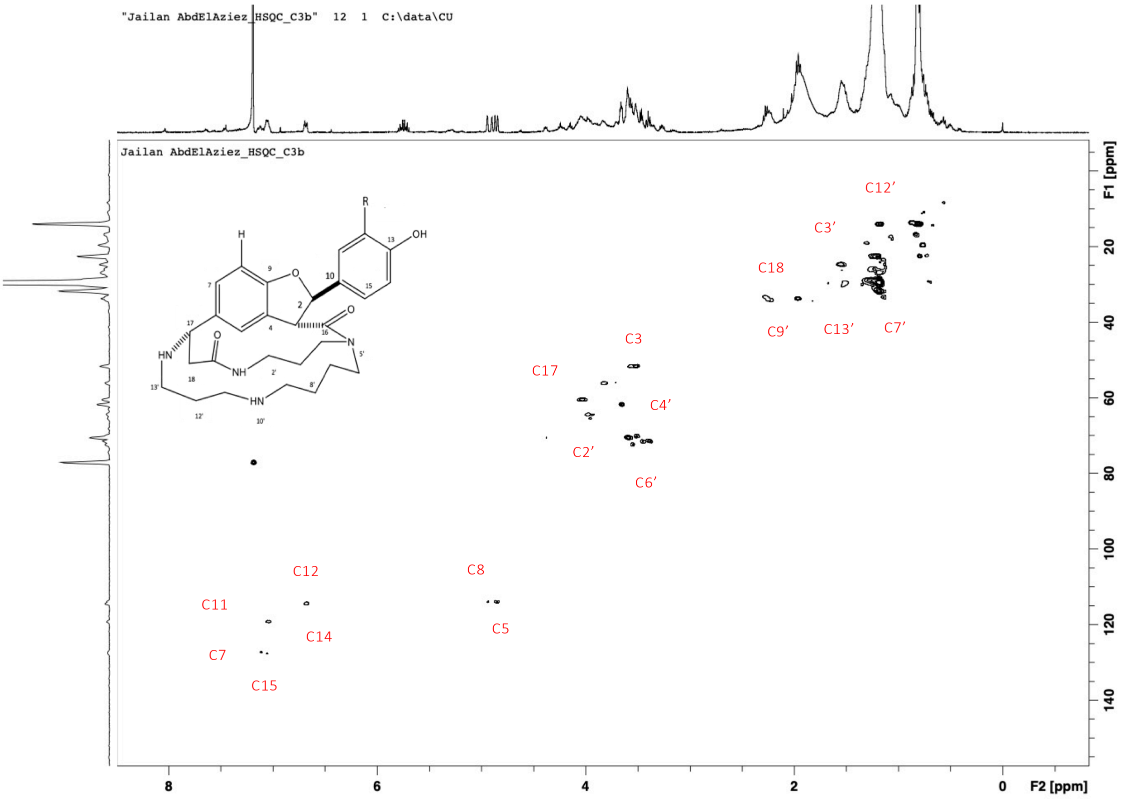

Figure S12: HSQC 2D-NMR Spectra of compound 1 (Ephedradine A).

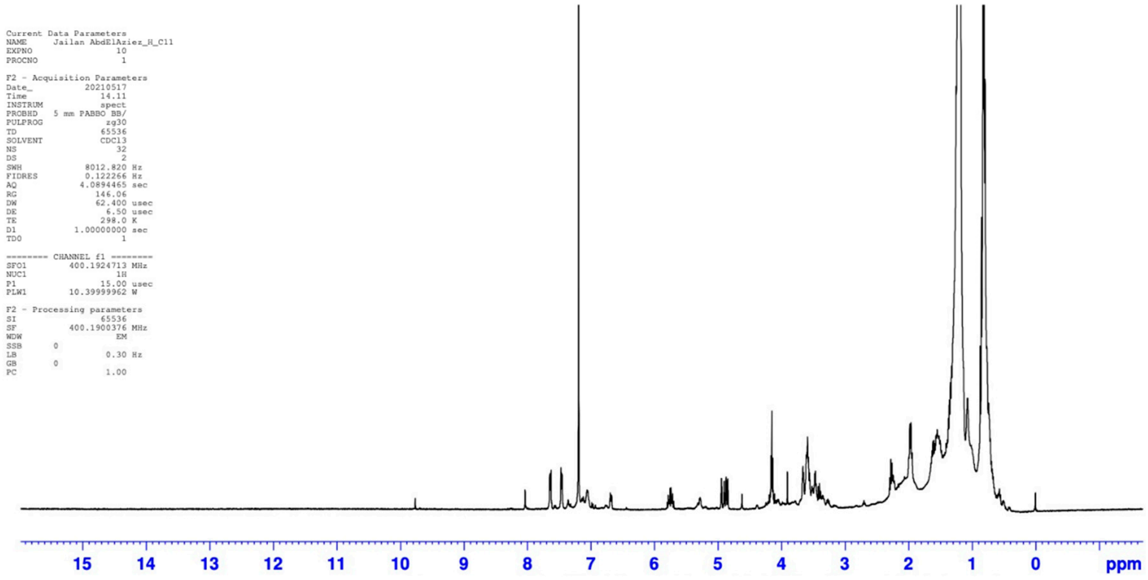

Figure S13: ^{1}HNMR Spectra of compound 2 (Ephedrannin Tr5).

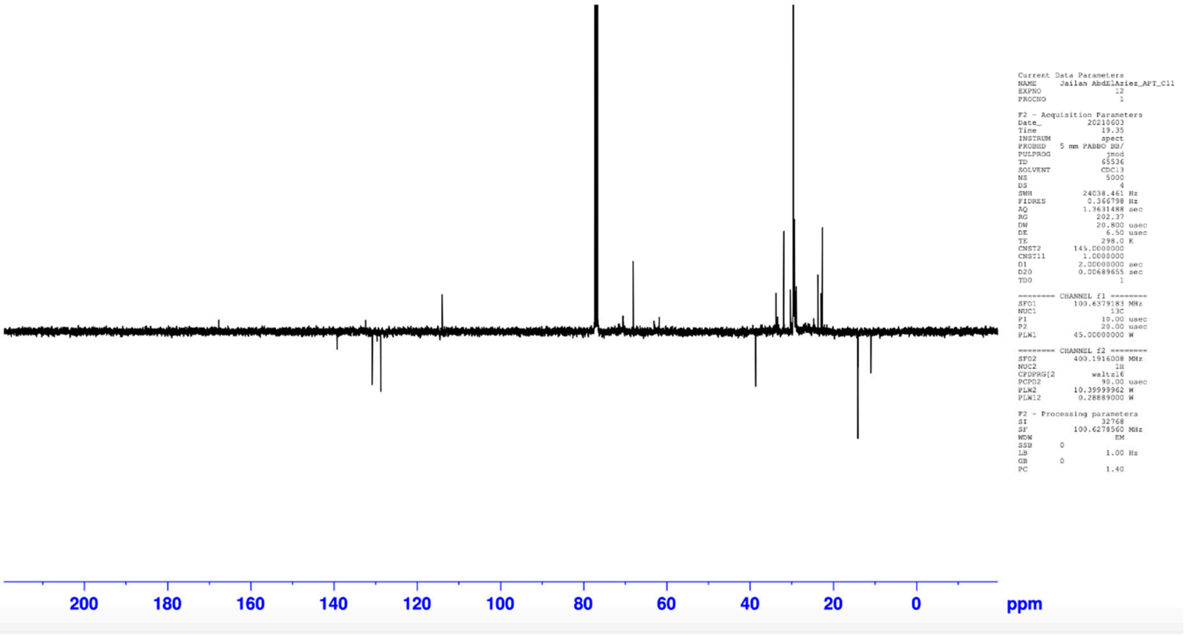

Figure S14: ^{13}C NMR Spectra of compound 2 (Ephedrannin Tr5).

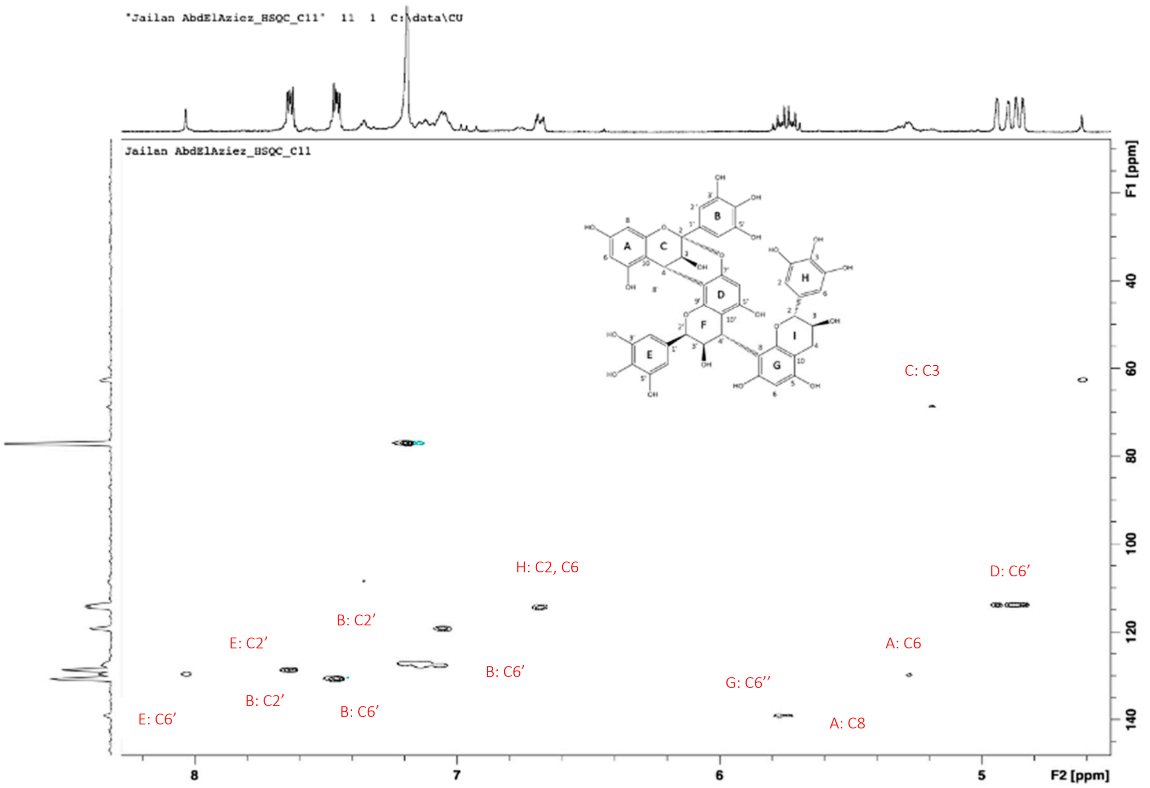

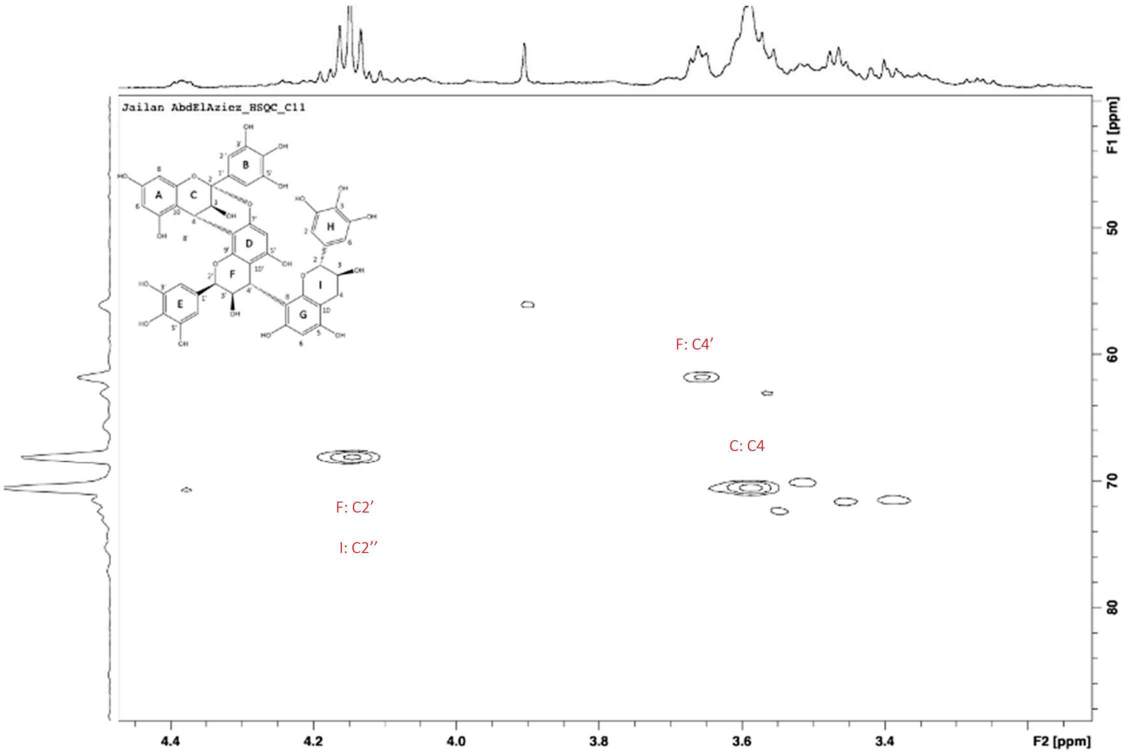

Figure S15: HSQC 2D-NMR Spectra of compound 2 (Ephedrannin Tr5).

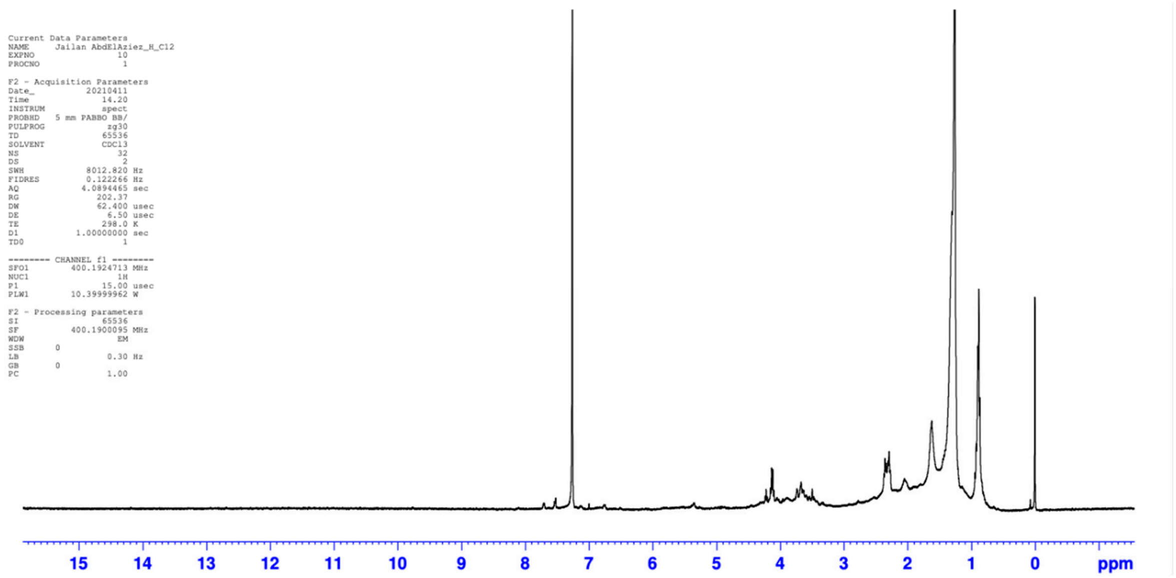

Figure S16: ^{1}HNMR Spectra of compound 3 (Nebrodenside).

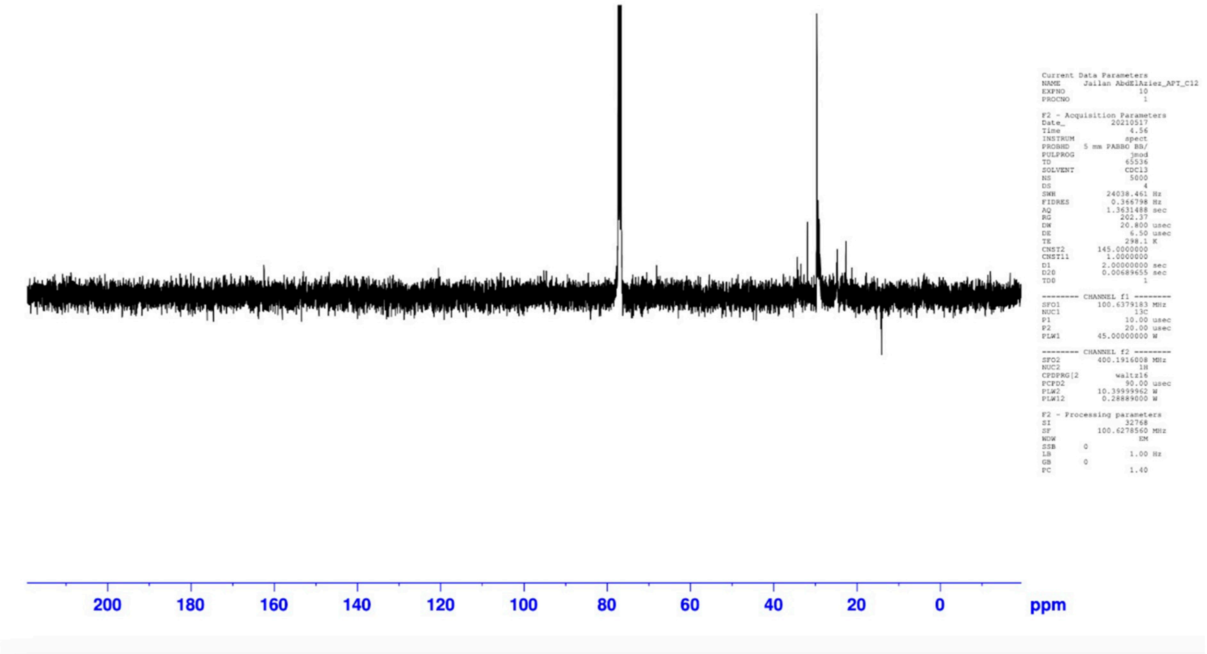

Figure S17: ^{13}C NMR Spectra of compound 3 (Nebrodenside).

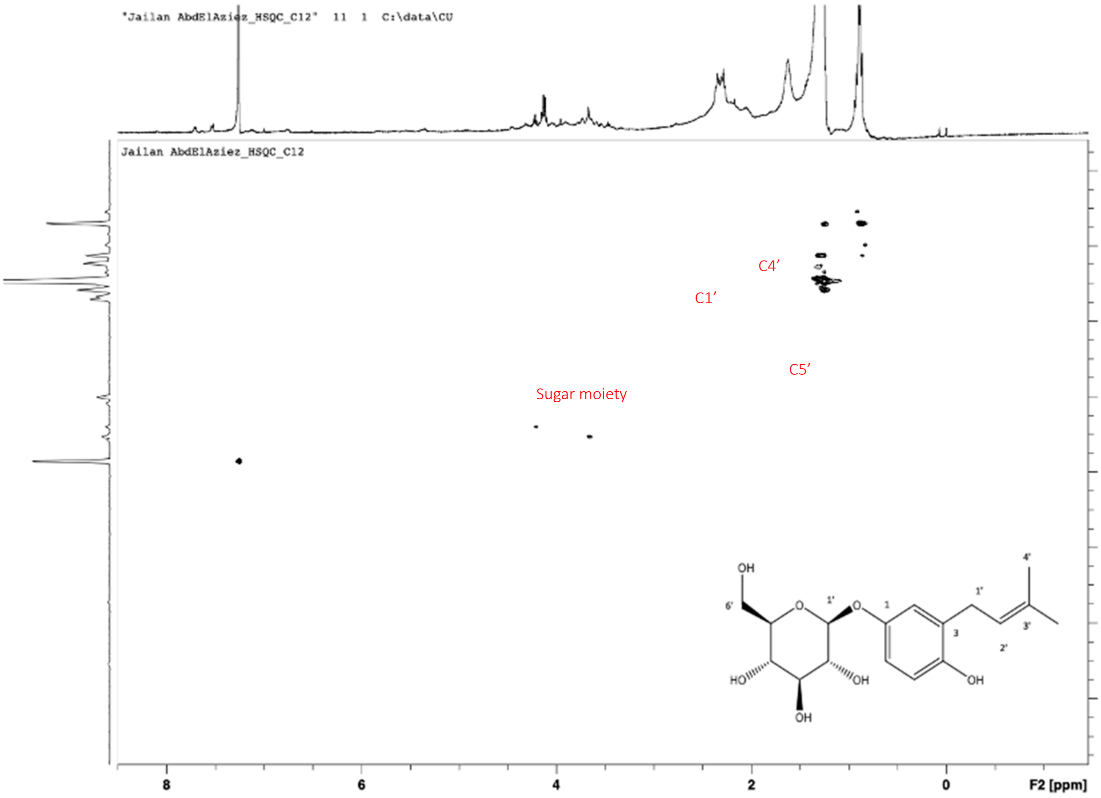
"Jailan AbdElAziez_HSQC_C12" 11 1 C:\data\CU
Jailan AbdElAziez_HSQC_C12
C4'
C1'
C5'
Sugar moiety
F1 [ppm]
F2 [ppm]
OH
6'
1'
O
1
3
1'
4'
3'
2'
HO
OH
OH
OH

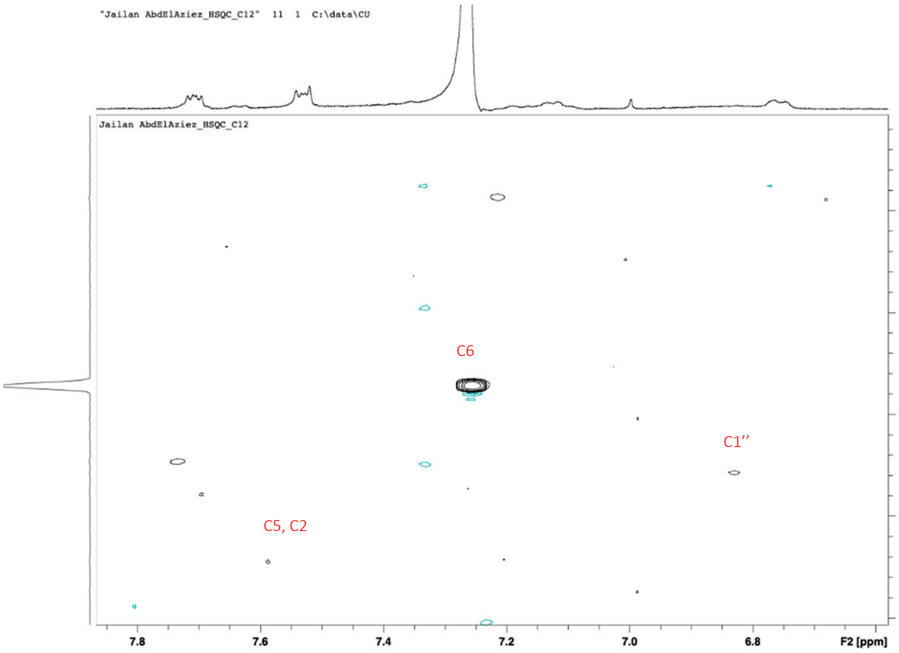
"Jailan AbdElAziez_HSQC_C12" 11 1 C:\data\CU
Jailan AbdElAziez_HSQC_C12
C6
C1''
C5, C2
F1 [ppm]
F2 [ppm]

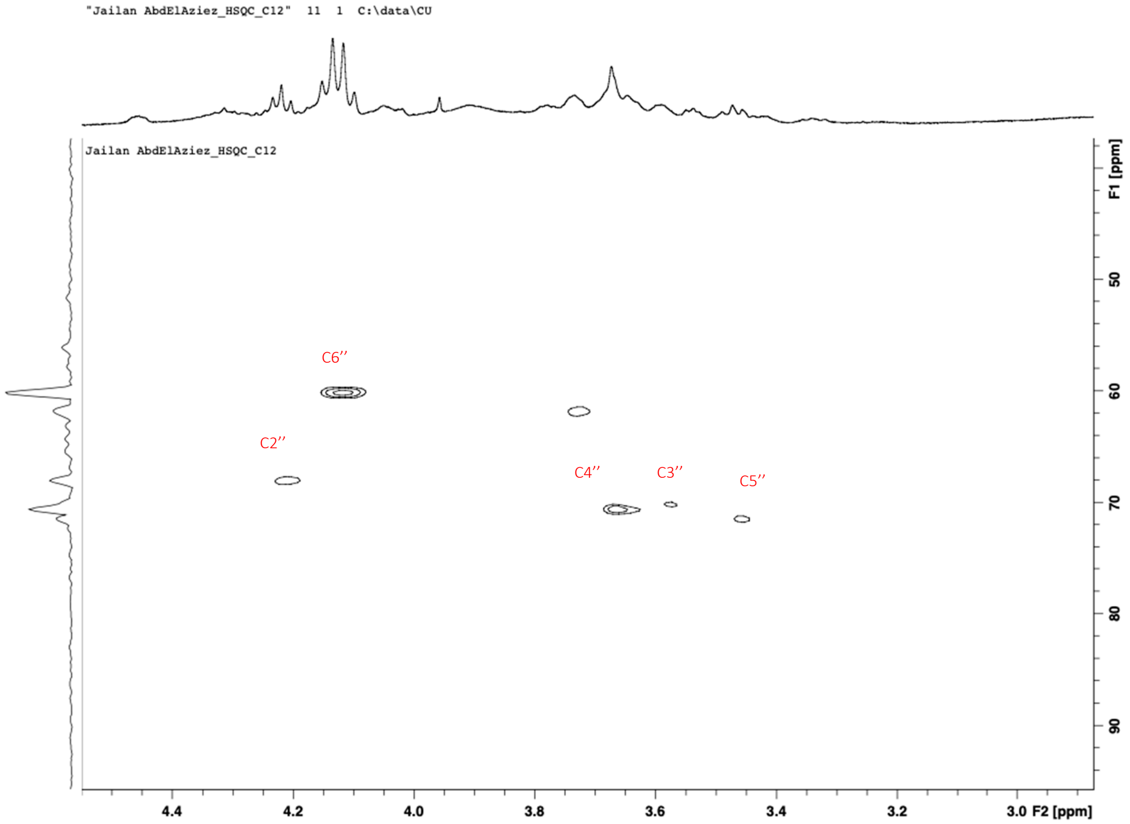

Figure S18: HSQC 2D-NMR Spectra of compound 3 (Nebrodenside).

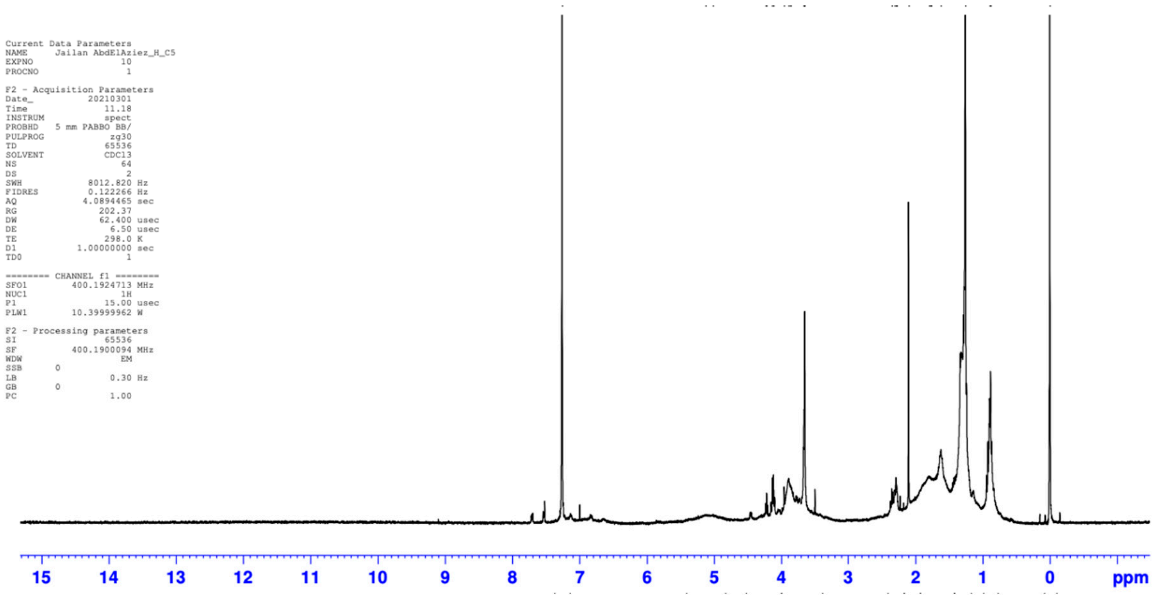

Figure S19: ^{1}HNMR Spectra of compound 4 (Ephedrannin B).

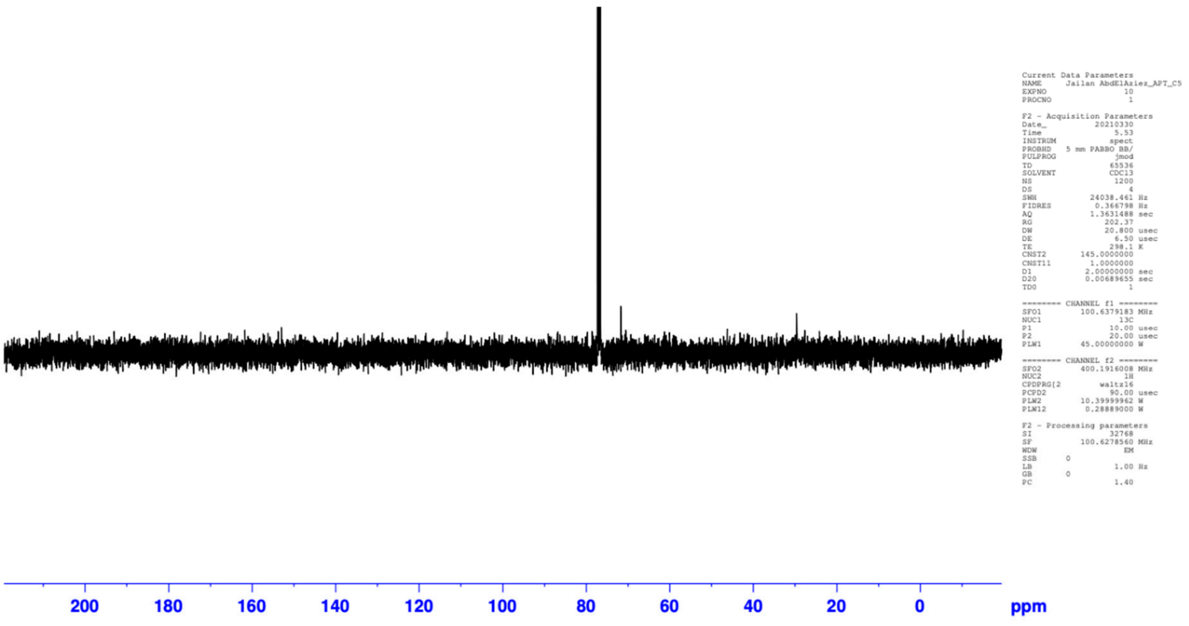

Figure S20: ^{13}C NMR Spectra of compound 4 (Ephedrannin B).

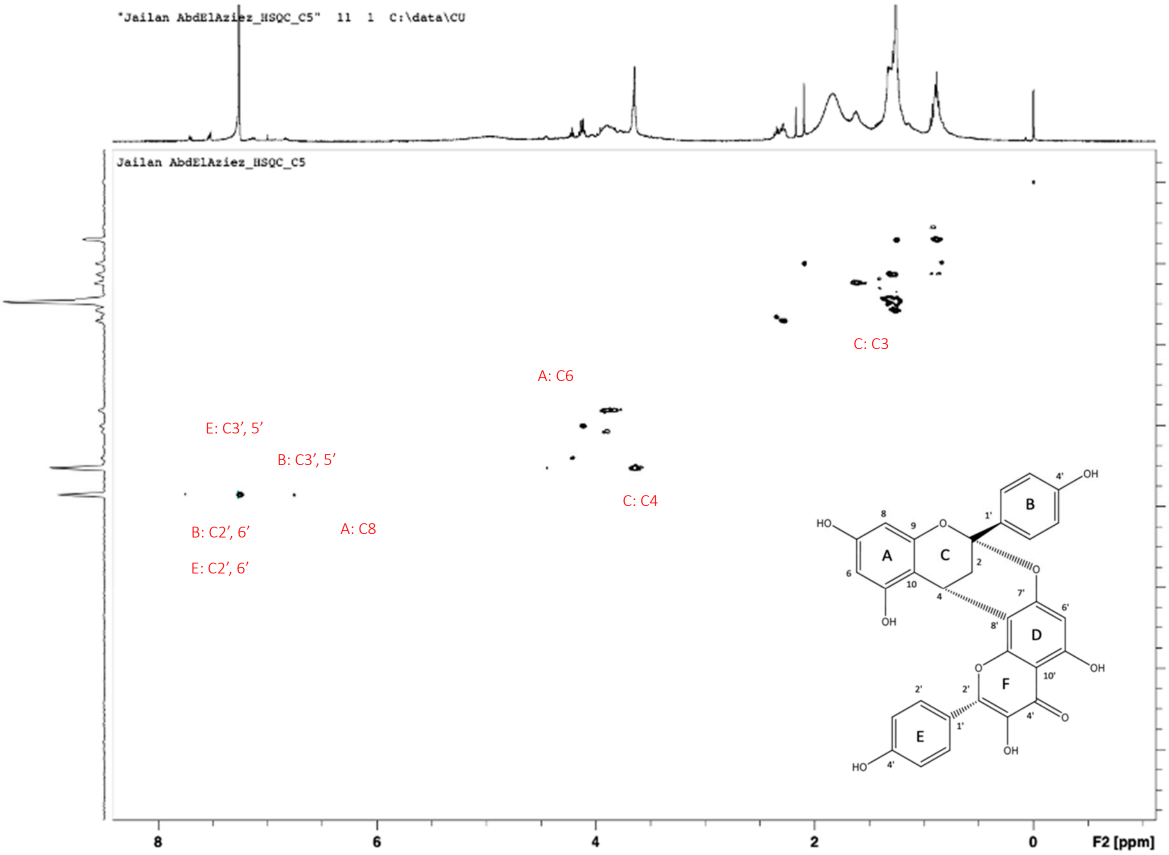

Figure S21: HSQC 2D-NMR Spectra of compound 4 (Ephedrannin B).

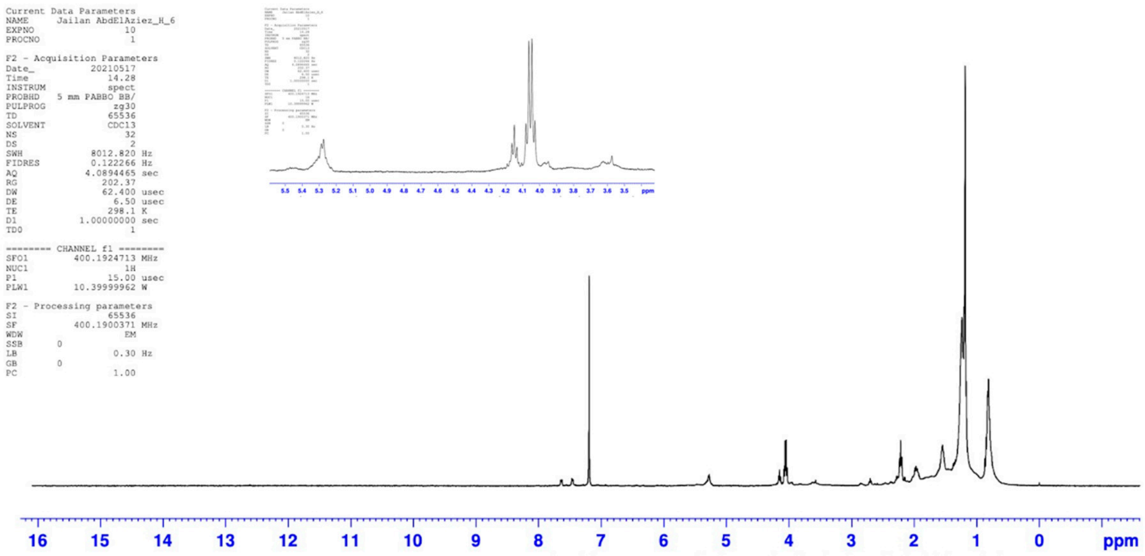

Figure S22: ^{1}HNMR Spectra of compound 5(Ephedrannin D1).

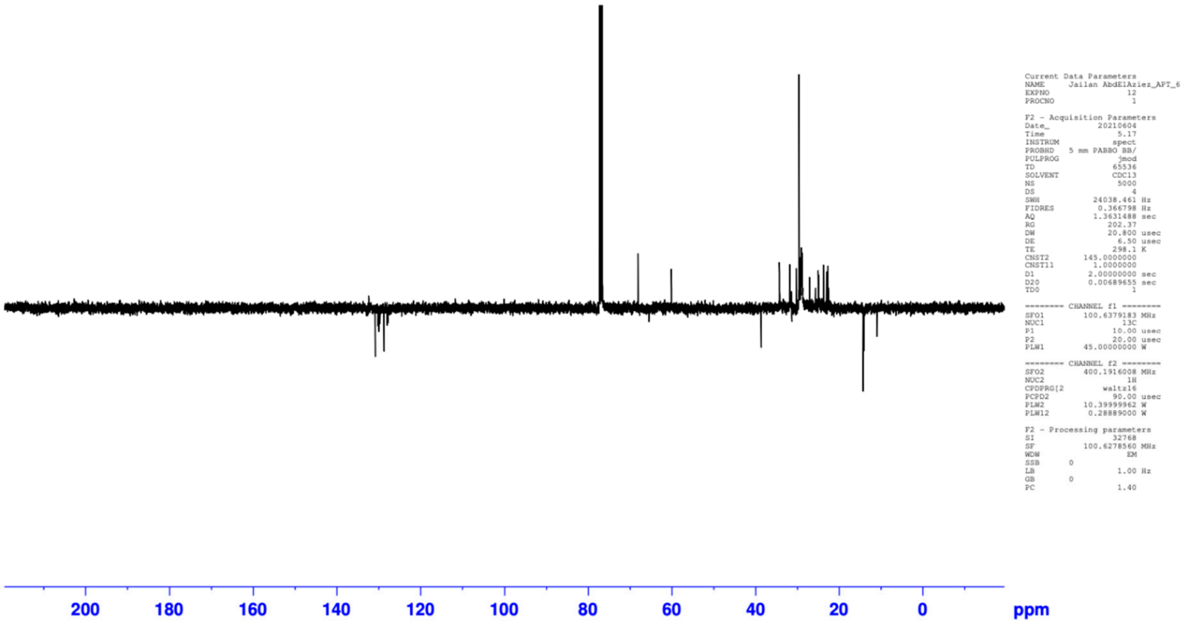

Figure S23: ^{13}C NMR Spectra of compound 5 (Ephedrannin D1).

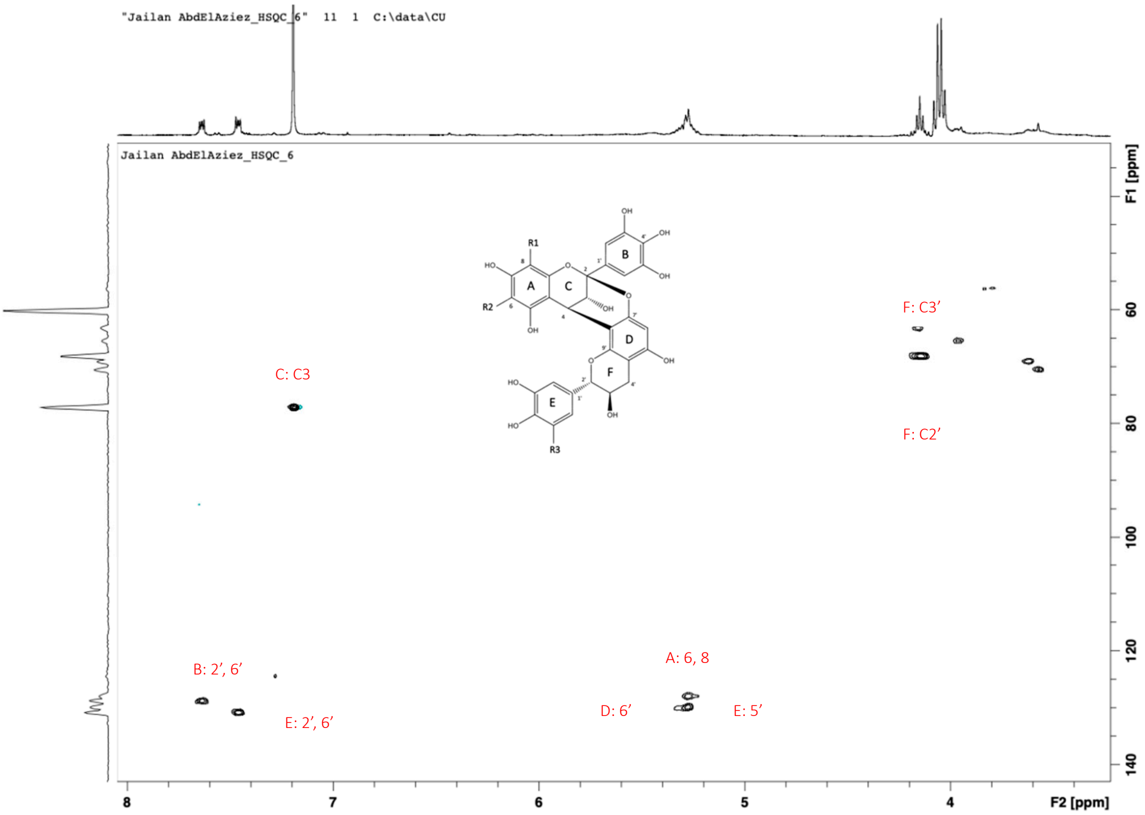

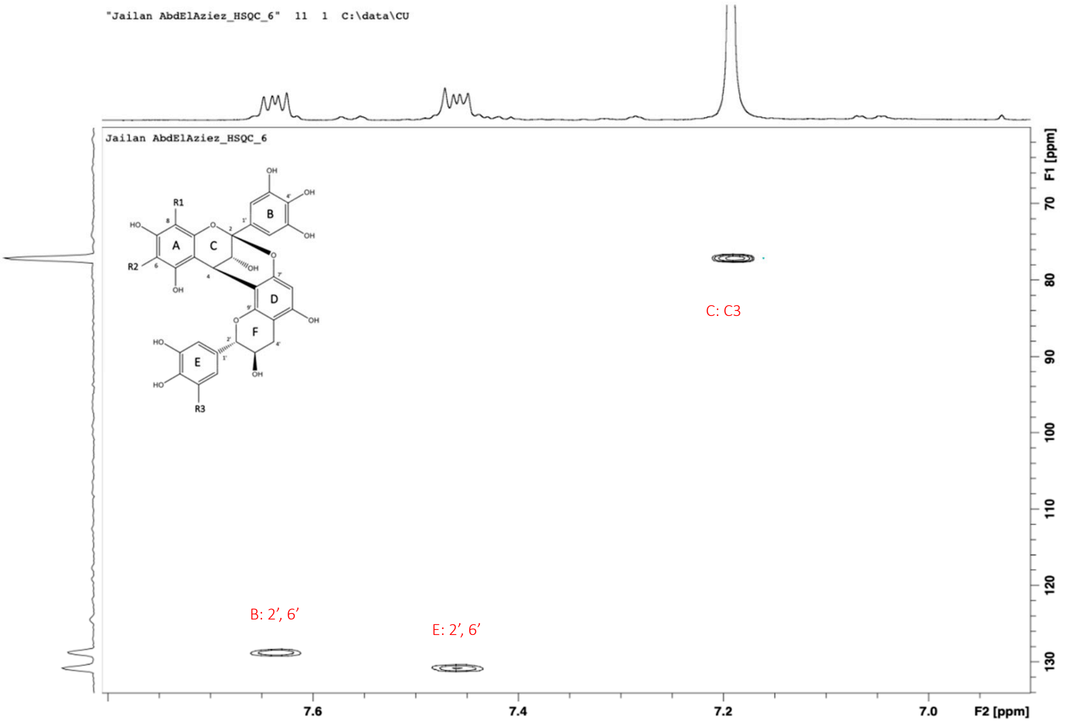

Figure S24: HSQC 2D-NMR Spectra of compound 5 (Ephedrannin D1).

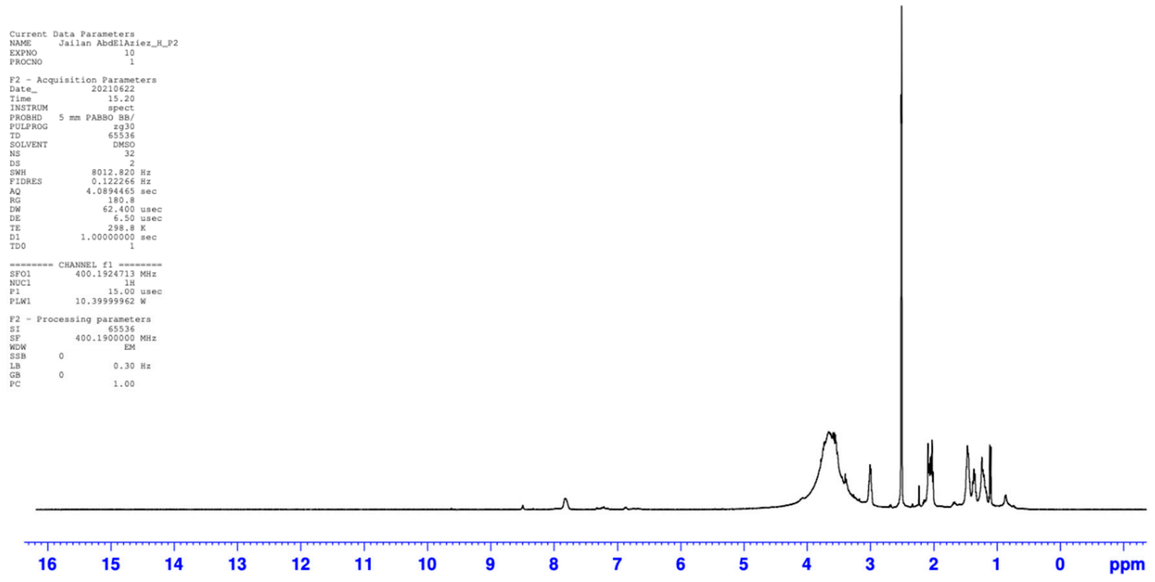

Figure S25: ^{1}HNMR Spectra of compound 6 (Adenosine).

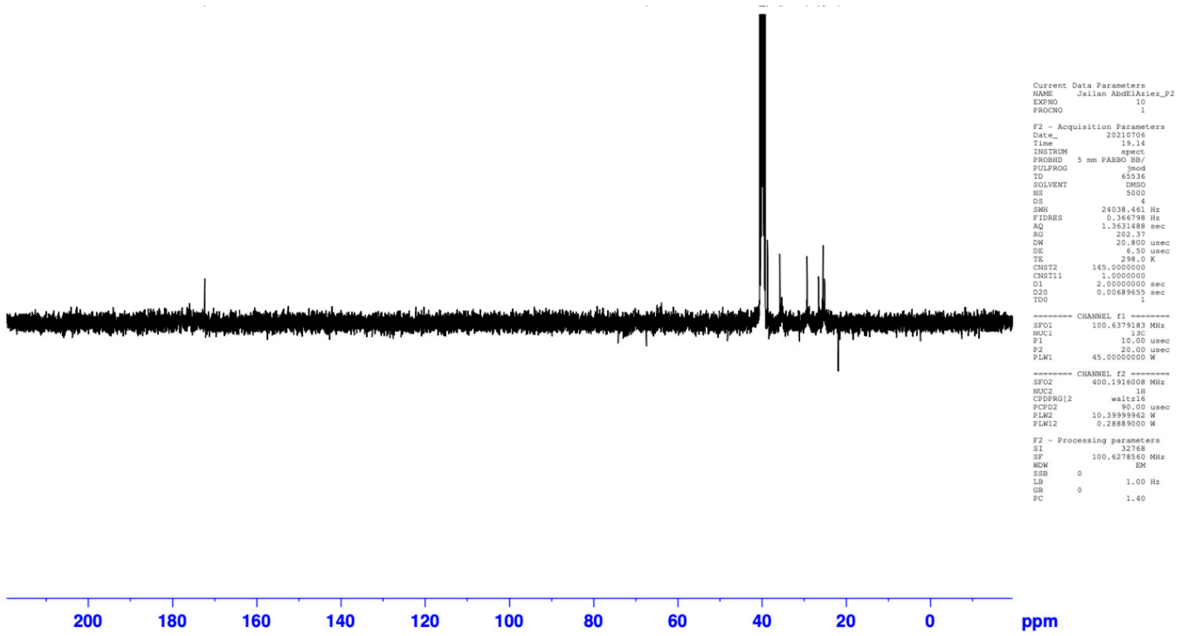

Figure S26: ^{13}C NMR Spectra of compound 6 (Adenosine).

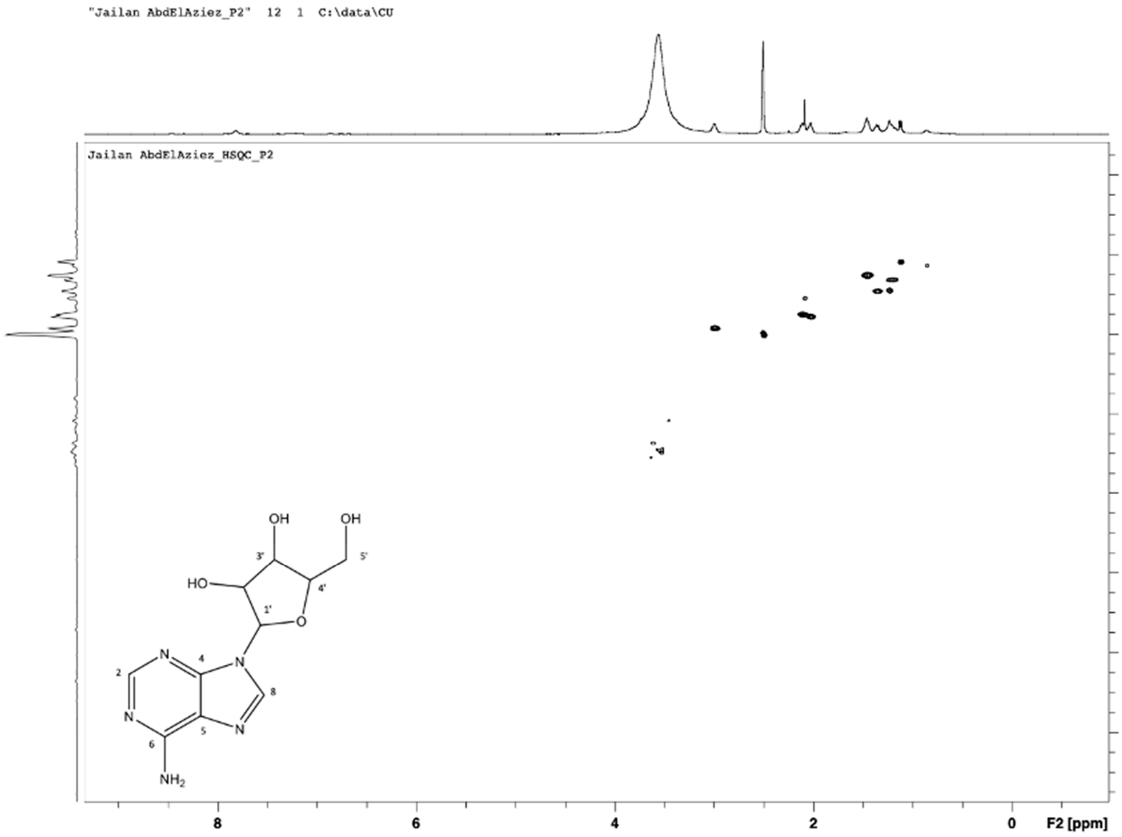

Figure S27: HSQC 2D-NMR Spectra of compound 6 (Adenosine).

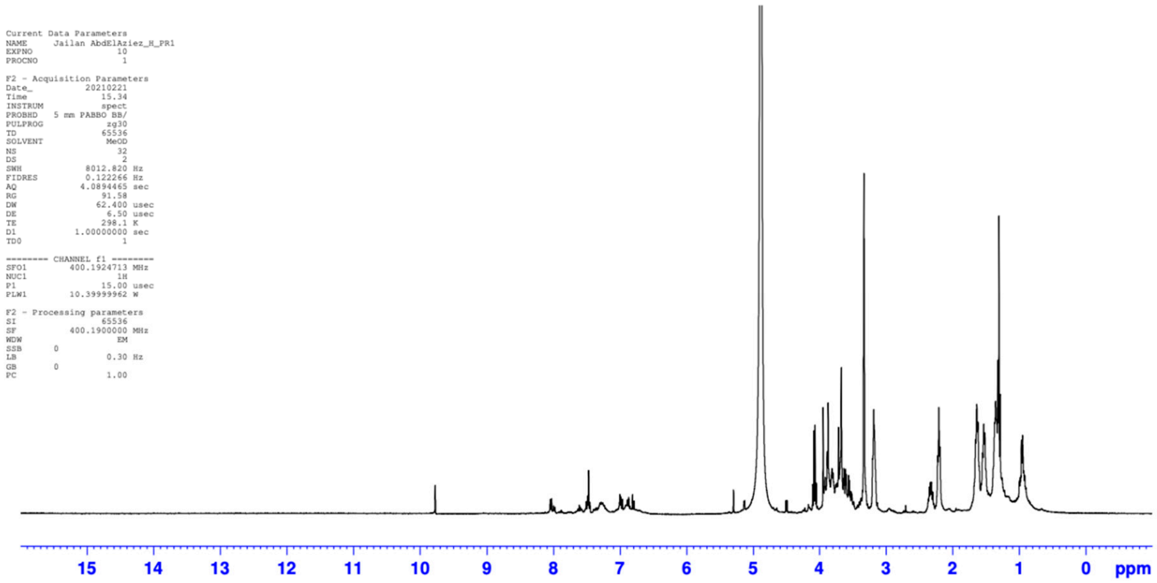

Figure S28: ^{1}HNMR Spectra of compound 7 (6- methoxyknurenic acid).

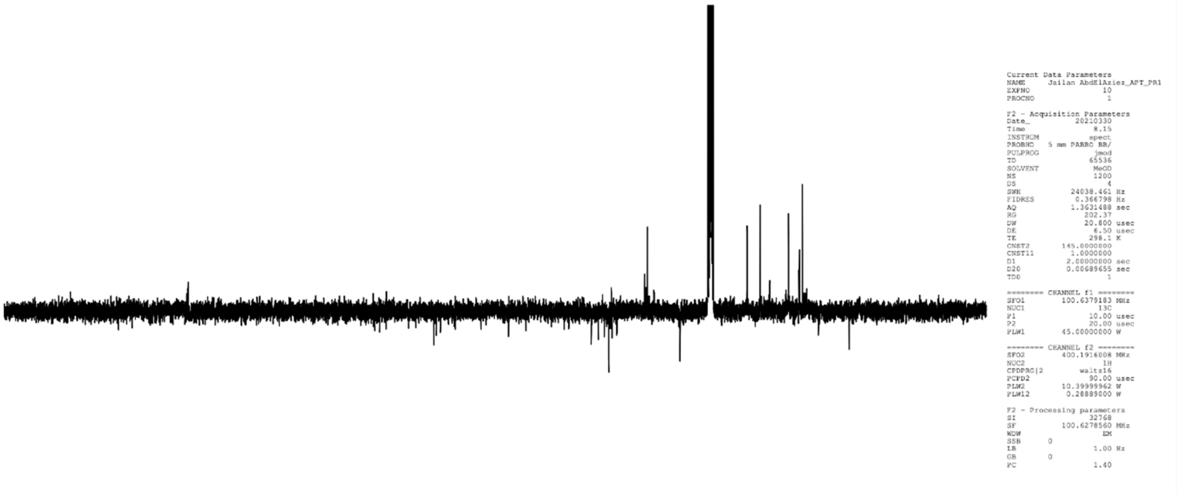

Figure S29: ^{13}C NMR Spectra of compound 7 (6- methoxyknurenic acid).

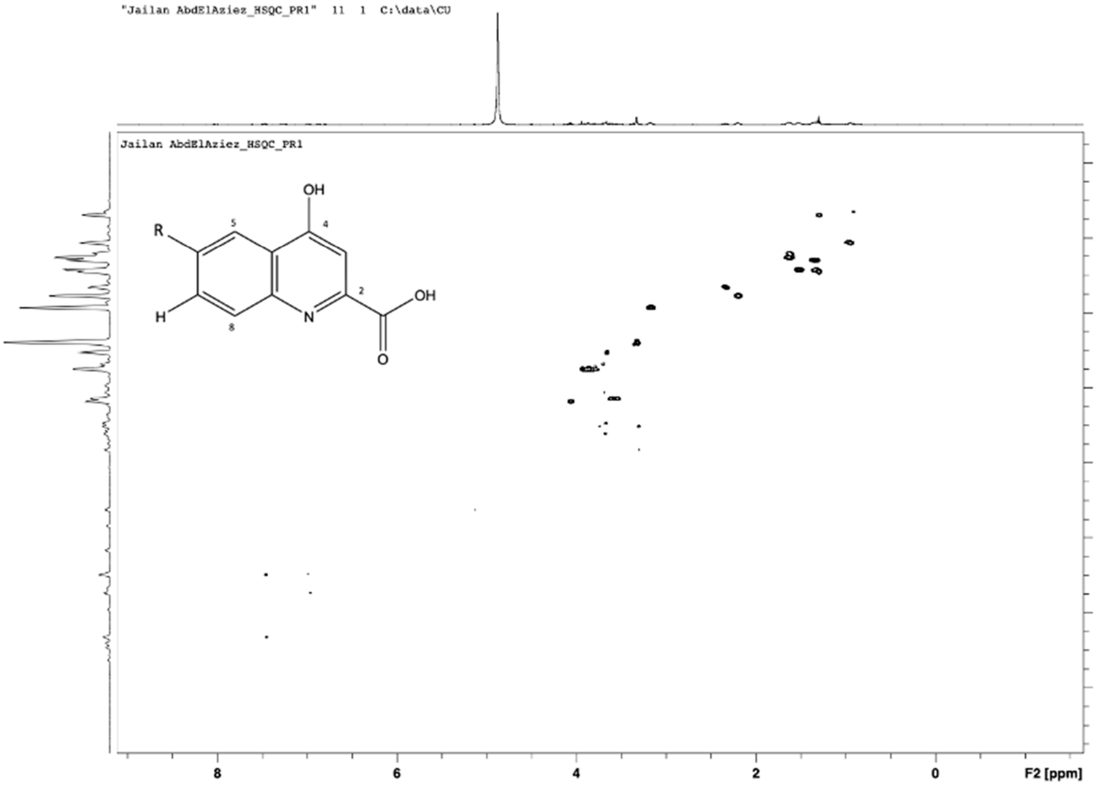

Figure S30: HSQC 2D-NMR Spectra of compound 7 (6- methoxyknurenic acid).

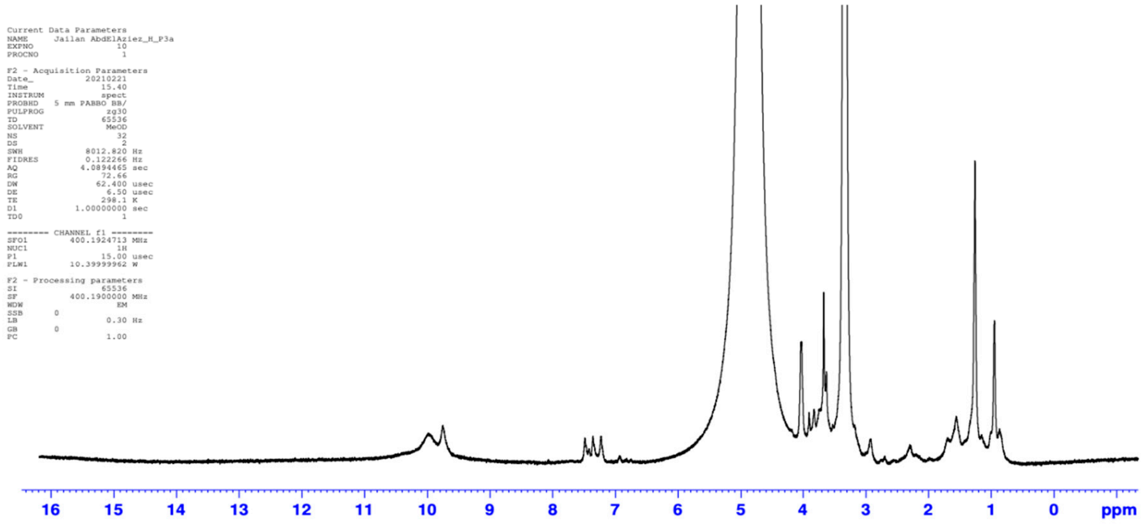

Figure S31: ^{1}HNMR Spectra of compound 8 (6-hydroxykynurenic acid).

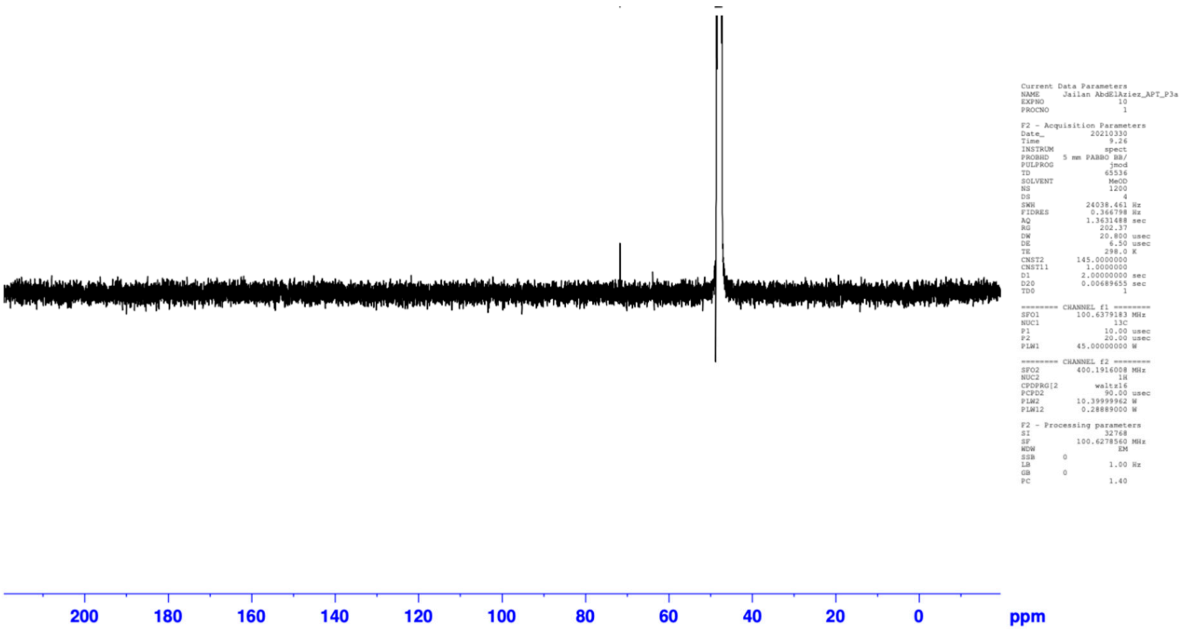

Figure S32: ^{13}C NMR Spectra of compound 8 (6-hydroxykynurenic acid).

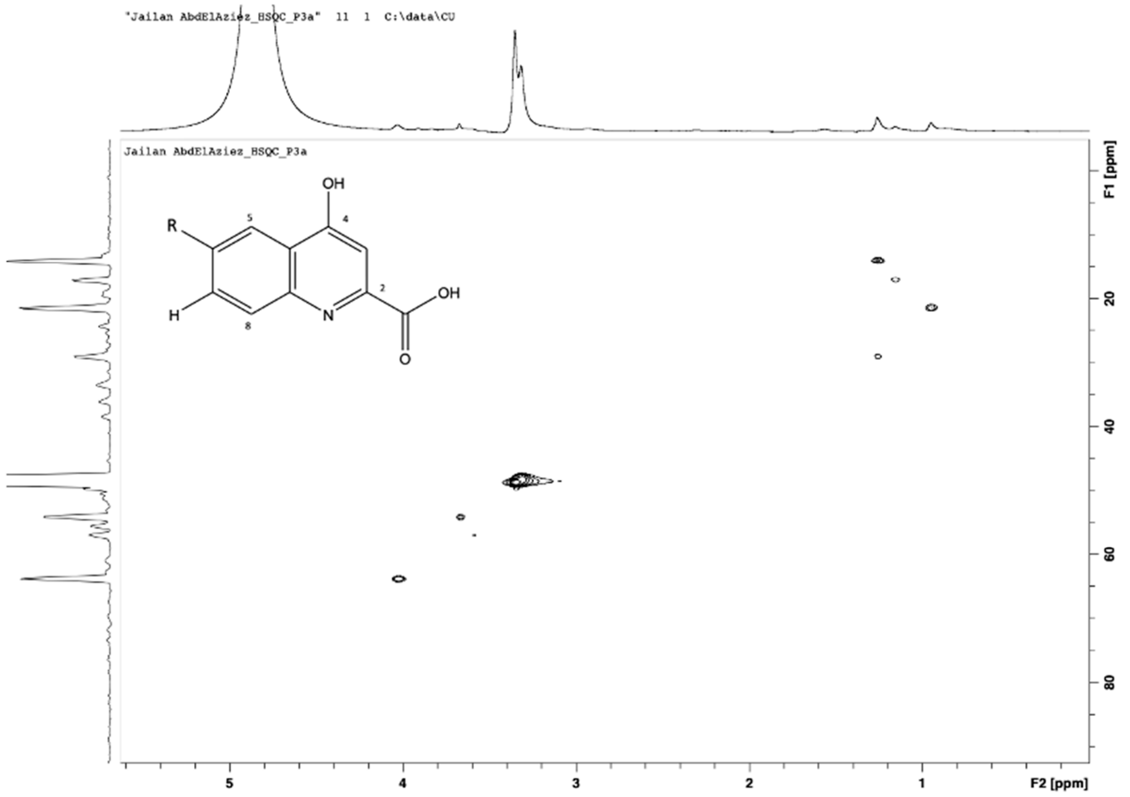

Figure S33: HSQC 2D-NMR Spectra of compound 8 (6-hydroxykynurenic acid).

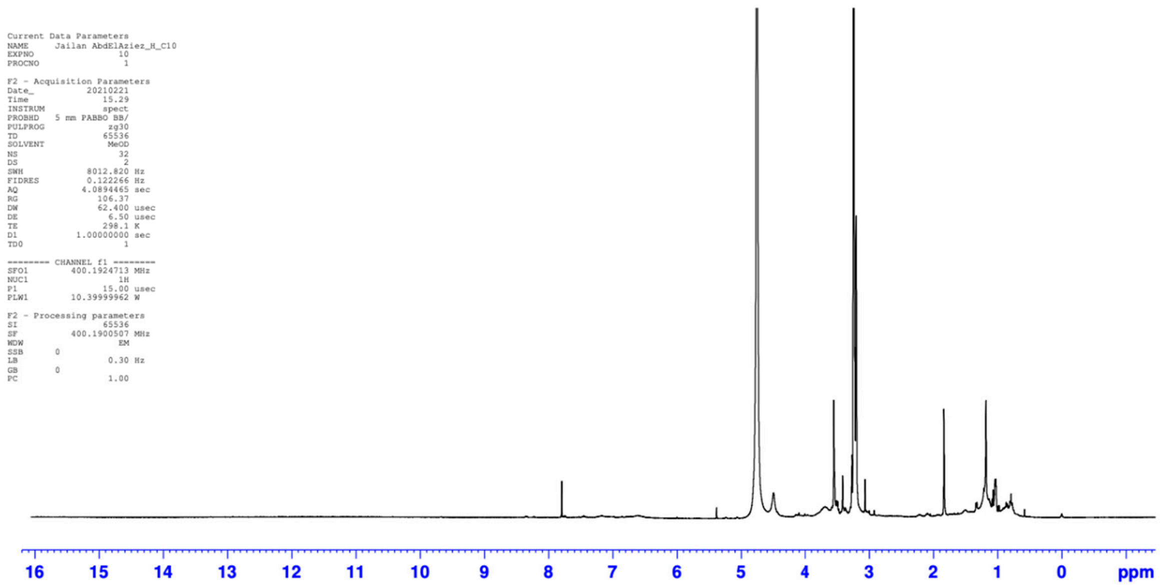

Figure S34: ^{1}HNMR Spectra of compound 9 (Ephedradine B).

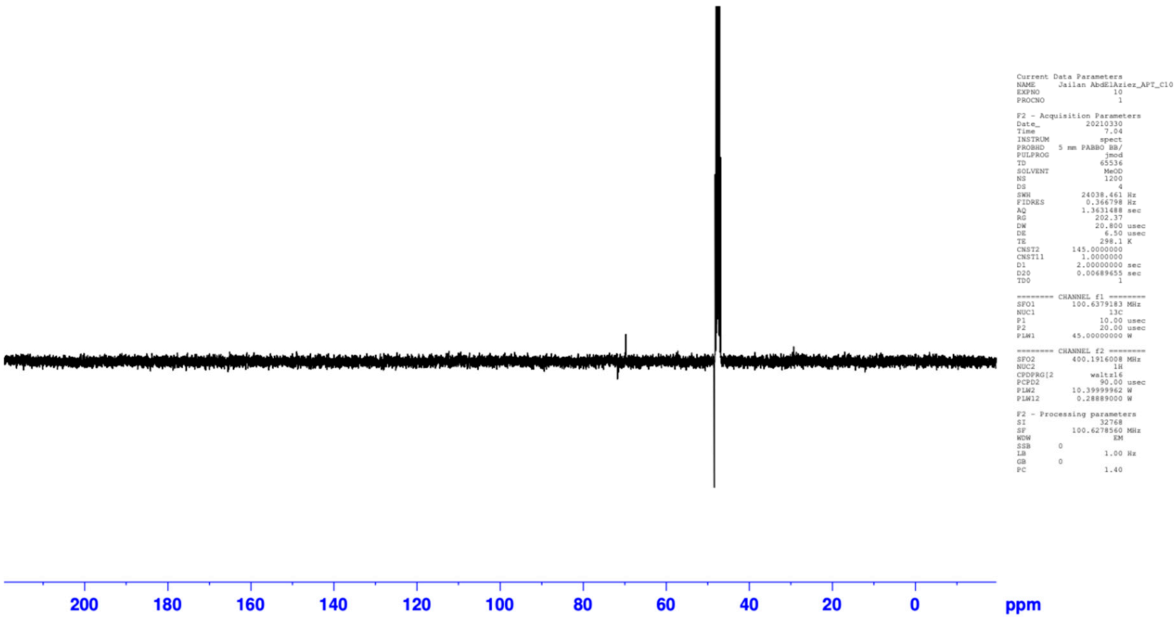

Figure S35: ^{13}C NMR Spectra of compound 9 (Ephedradine B).

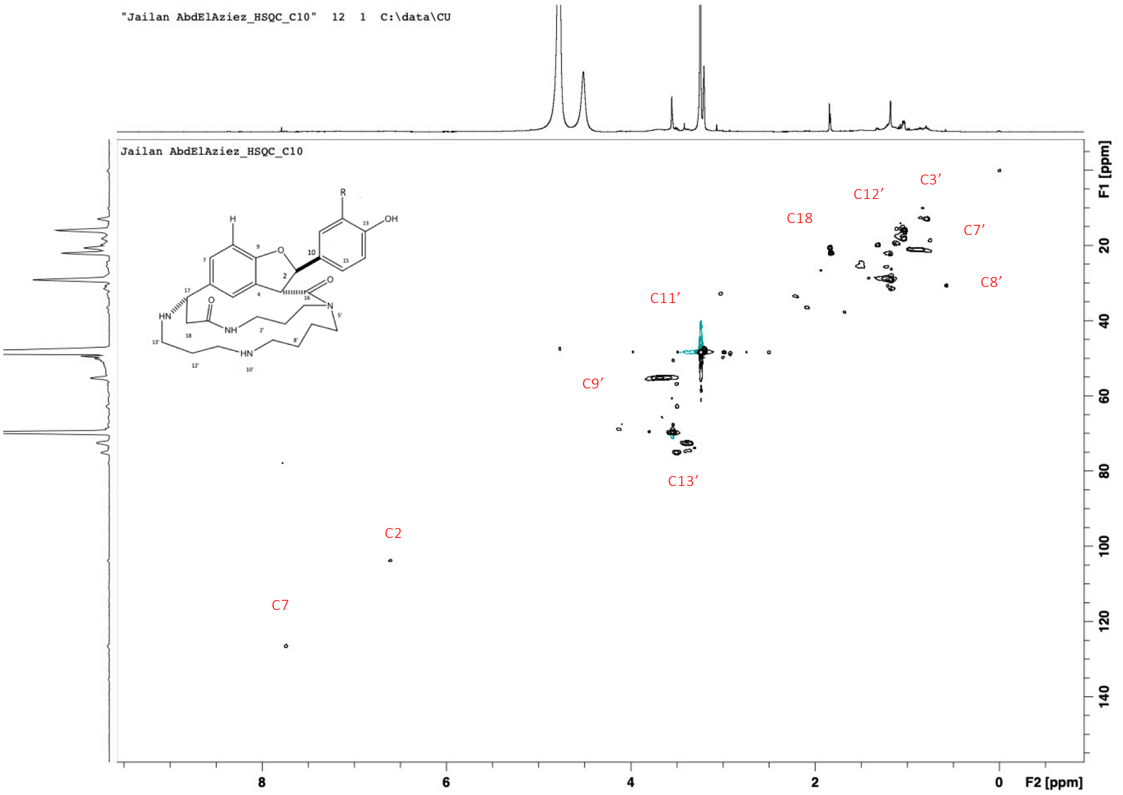
"Jailan AbdElAziez_HSQC_C10" 12 1 C:\data\CU
Jailan AbdElAziez_HSQC_C10
C3'
C12'
C18
C7'
C8'
C11'
C9'
C13'
C2
C7
F1 [ppm]
F2 [ppm]

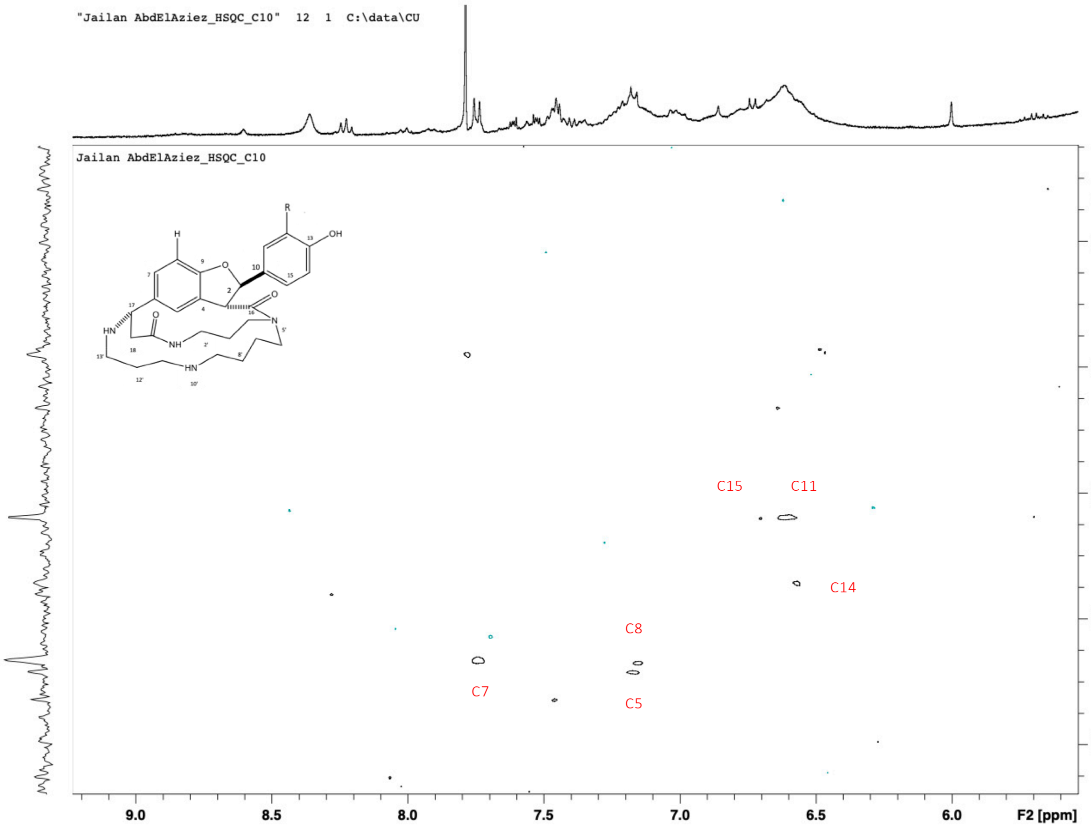
"Jailan AbdElAziez_HSQC_C10" 12 1 C:\data\CU
Jailan AbdElAziez_HSQC_C10
C15
C11
C14
C8
C7
C5
F1 [ppm]
F2 [ppm]

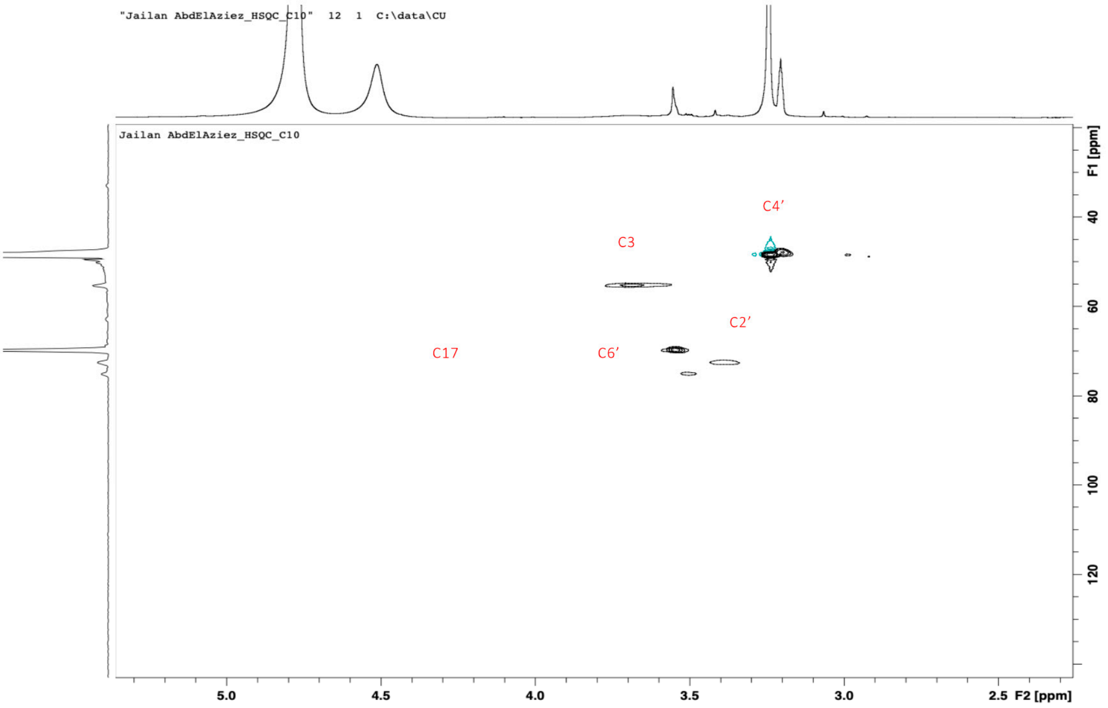

Figure S36: HSQC 2D-NMR Spectra of compound 9 (Ephedradine B).
